# A conformation-specific nanobody targeting the nicotinamide mononucleotide-activated state of SARM1

**DOI:** 10.1038/s41467-022-35581-y

**Published:** 2022-12-22

**Authors:** Yun Nan Hou, Yang Cai, Wan Hua Li, Wei Ming He, Zhi Ying Zhao, Wen Jie Zhu, Qiang Wang, Xinyi Mai, Jun Liu, Hon Cheung Lee, Goran Stjepanovic, Hongmin Zhang, Yong Juan Zhao

**Affiliations:** 1 State Key Laboratory of Chemical Oncogenomics, Key Laboratory of Chemical Genomics, Peking University Shenzhen Graduate School, Shenzhen, 518055 China; 2 Department of Biology, School of Life Sciences, Southern University of Science and Technology, Shenzhen, 518055 China; 3 Ciechanover Institute of Precision and Regenerative Medicine, School of Medicine, The Chinese University of Hong Kong, Shenzhen, 518172 China; 4 Kobilka Institute of Innovative Drug Discovery, School of Medicine, The Chinese University of Hong Kong, Shenzhen, 518172 China

**Keywords:** Cryoelectron microscopy, Nucleotide-binding proteins, Antibody generation, Enzyme mechanisms

## Abstract

Sterile alpha (SAM) and Toll/interleukin-1 receptor (TIR) motif containing 1 (SARM1) is an autoinhibitory NAD-consuming enzyme that is activated by the accumulation of nicotinamide mononucleotide (NMN) during axonal injury. Its activation mechanism is not fully understood. Here, we generate a nanobody, Nb-C6, that specifically recognizes NMN-activated SARM1. Nb-C6 stains only the activated SARM1 in cells stimulated with CZ-48, a permeant mimetic of NMN, and partially activates SARM1 in vitro and in cells. Cryo-EM of NMN/SARM1/Nb-C6 complex shows an octameric structure with ARM domains bending significantly inward and swinging out together with TIR domains. Nb-C6 binds to SAM domain of the activated SARM1 and stabilized its ARM domain. Mass spectrometry analyses indicate that the activated SARM1 in solution is highly dynamic and that the neighboring TIRs form transient dimers via the surface close to one BB loop. We show that Nb-C6 is a valuable tool for studies of SARM1 activation.

## Abstract (web summary)

SARM1 is a key player in axon degeneration. Here, the authors generate a nanobody, which specifically recognizes the NMN-bound state of SARM1 and helps resolve the SARM1 structure in an intermediate state of activation.

## Introduction

Axon degeneration (AxD) is regarded as an early event in various neurodegenerative diseases including amyotrophic lateral sclerosis, multiple sclerosis, and Parkinson’s disease^1^. AxD is a programmed process regulated by the axonal NAD levels. This is revealed by the finding that *Wld*^*s*^ mice expressing cytosolic NMNAT1 delayed the axonal NAD depletion after injury and reduced AxD dramatically^2,3^. Genetic screening in *Drosophila melanogaster* led to the discovery of the crucial protein, i.e., sterile alpha (SAM) and Toll/interleukin-1 (TIR) motif–containing 1 (SARM1), the ablation of which also significantly delayed AxD^4^.

SARM1 is a multidomain protein composed of an N-terminal 27-aa mitochondrial localization signal, an Armadillo repeat motif (ARM) domain, a SAM domain, and a C-terminal TIR domain. Truncation studies suggest that TIR is the catalytic NADase domain, while ARM serves an autoinhibitory function^5–7^. The enzymatic activity of the autoinhibited SARM1 is activated by nicotinamide mononucleotide (NMN) in vitro or by CZ-48 in live cells, a permeant mimetic of NMN we previously developed^8^. In addition to being a substrate of SARM1, NAD has also been found to be also an endogenous inhibitor^9,10^, and the increased NMN/NAD ratio actually regulates SARM1 activation and AxD^11^. These findings established a working model of AxD, in which NMN accumulation and NAD decrease, caused by the rapid degradation of NMNAT2 after axon injury^12,13^, activating SARM1. The result is the depletion of NAD, leading to AxD.

SARM1 is, in fact, a signaling enzyme possessing multicatalytic functions. In addition to its NADase activity, it also catalyzes the production of two calcium-mobilizing messengers, cyclic ADP-ribose (cADPR) and nicotinic acid adenine dinucleotide phosphate (NAADP)^8,14–16^. In paclitaxel-induced peripheral neuropathy, SARM1 is activated to produce cADPR, which in turn elevates cellular calcium, resulting in AxD^17^. SARM1 is mainly expressed in neurons^18^ but is also found in a variety of cells^8^. It is mainly localized in the outer membrane of mitochondria with the catalytic domain facing the cytosol^19^ and has ready access to the substrate NAD. All these features, together with its autoregulatable nature, make SARM1 a potential calcium signaling enzyme with roles not only in AxD^20^ but also potentially other physiological conditions.

SARM1 was originally thought to function as a dimer^5,6,8^ until the octameric structure of its SAM domain was shown^21^. Shortly afterwards, several structures of SARM1 in its inactive form were solved by cryo-EM^9–11,22,23^. In these structures, the ARM domains form the outer periphery of the octameric ring with the TIR domains wedged in between, while SAM domains form the inner periphery around the center hole of the ring^10^. The binding site of NMN at the ARM domain has been revealed by the crystal structure of the isolated domain from *Drosophila* SARM1, i.e., dSARM, whose occupation can induce a significant conformational change in the entire domain^11^. Despite much effort, the structure of the NMN-activated form of SARM1 has not been fully determined, largely due to the flexibility of the ARM and TIR domains upon activation. Recently, Shi and his colleagues discovered an NAD derivative, 1AD, which stabilizes the oligomerization of the TIR domain and enables the resolution of the active site and ARM-SAM^NMN^ conformations by a combination of crystallization and cryo-EM techniques^24^. A model for SARM1 was built based on these structures. NMN binds to the allosteric site of ARM and causes its inward bending, which lifts the ARM domain through the rigid ARM-SAM linker. The lifted and rotated ARM clashes with the neighboring TIR domain, which may lead to the release of TIR domains and constitution of the active pocket.

In this study, we generated a unique nanobody, Nb-C6, that binds specifically to the active form of SARM1 and stabilizes it; thus, it could stain and quantify the activated SARM1 and facilitate the activation of SARM1 in vitro and in cells after a longer incubation. Nb-C6 also allowed the structural determination of NMN-activated full-length SARM1. Detailed analyses indicate that binding and activation by NMN induces large conformational changes in octameric SARM1, which is largely consistent with previous observations^11,24^. The HDX-MS and XL-MS data confirmed the conformational changes upon activation and further indicated the heterogenicity of the NMN conformations in solution. Together, our work not only provides a valuable tool to visualize active SARM1 and to activate SARM1 in cells, but also sheds light on the activation mechanism of SARM1. NMN binding causes inward bending of ARM, which stretches the linker between ARM and SAM domains and consequently leads to the dissociation of ARM from SAM and exposes the binding site of Nb-C6. Another consequence of ARM bending is weakening of the association between ARM and TIR domains, which facilitates the oligomerization of TIR and the formation of the active pocket.

## Results

### Generation and isolation of a nanobody that specifically recognizes NMN-activated SARM1

**Fig. 1:**
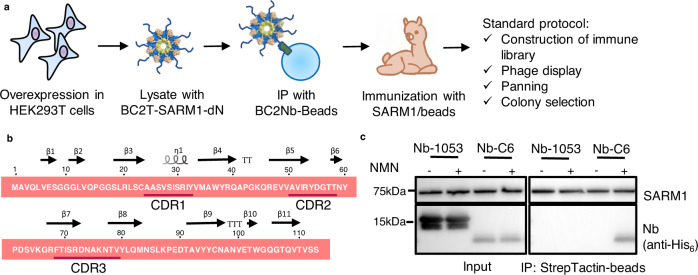
Nanobody C6 binds to the NMN-activated SARM1. **a** The process of Nb-C6 development. **b** The primary sequence and secondary structure of Nb-C6. **c** Nb-C6 binds to the NMN-activated SARM1 determined by the pulldown assay. The cell lysates containing the recombinant SARM1 were incubated with the StrepTactin^TM^ beads, together with 200 ng/mL Nb-C6, or a CD38 nanobody Nb-1053^26^ as a control, in the presence or absence of 100 μM NMN. The protein complex was eluted by 2 mM biotin and analyzed by western blots (*n* = 3 biological independent experiments). Source data are provided as a Source Data file.

Our approach to determining the structure of the active form of SARM1 was to stabilize it using a nanobody (Nb). We produced recombinant dN-SARM1, with the N-terminal 27-aa segment truncated and carrying a BC2-tag^25^, in HEK293T cells. The proteins immunoprecipitated by beads conjugated with a BC2 nanobody^25^ were mixed with Freund’s adjuvant and used to immunize an alpaca. After several rounds of immunization, the nanobody library was constructed from the mRNAs of the peripheral lymphocytes. Phage display and clone selection were performed as described in the Methods section (Fig. 1a). Several nanobodies were cloned, expressed in bacteria and purified with a His_6_-tag affinity column using the protocol described previously^26^. One of the nanobodies, Nb-C6, the sequence and secondary structure of which are shown in Fig. 1b, was selected because it recognized mainly the NMN-activated SARM1. In the pull-down assay shown in Fig. 1c, Nb-C6 coprecipitated with SARM1 mainly in the presence of the activator NMN, while Nb-1053^26^, an irrelevant Nb control, did not bind SARM1, indicating that Nb-C6 is specific for the activated conformation.

### Visualization and in vivo activation of SARM1 using Nb-C6

**Fig. 2:**
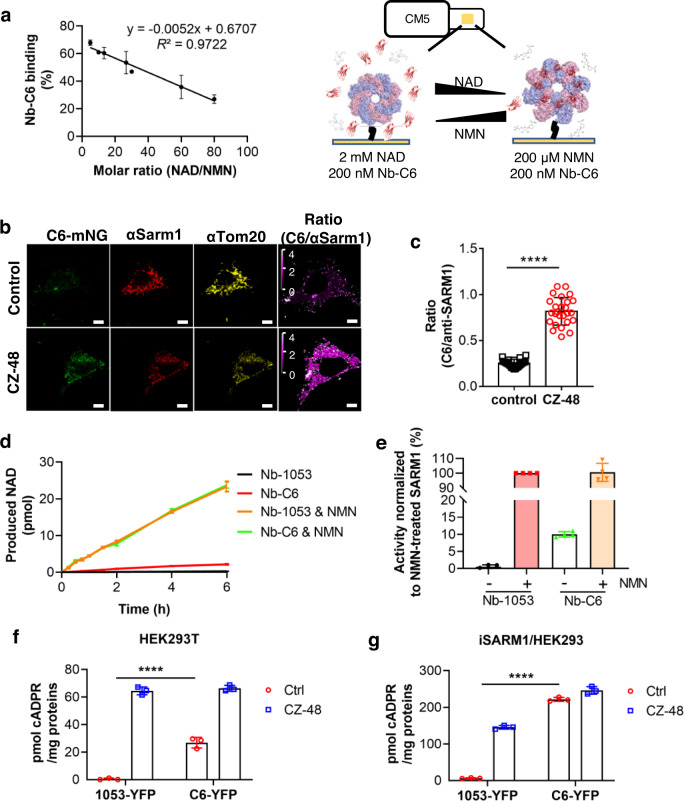
Nb-C6 indicates and facilitates SARM1’s activation in vitro and in cells. **a** SPR analysis of Nb-C6 binding to the immobilized SARM1 when the mobilizing modulator switching from 2 mM NAD to 200 μM NMN. The Nb-C6 binding, calculated from RU value and normalized with that in the condition of 200 μM NMN (set as 100%), and the molar ratio of NAD and NMN were fit into a linear model. (*n* = 4 biologically independent experiments, mean ± SD) **b** Nb-C6 stains the CZ-48-activated SARM1. HEK293 cells carrying the inducible expression cassette for SARM1 were treated with 1 μg/mL doxycycline for 12 h and then 100 μM CZ-48 for 6 h. The cells were fixed and stained with C6-mNeonGreen, anti-SARM1, anti-Tom20 as described in the Methods. Horizontal scale bar: 5 μm; vertical scale bar: 0-4, ratio of the signals from Nb-C6 staining and anti-SARM1 staining. **c** The ratio of Nb-C6 and anti-SARM1 signals was calculated and plotted. (*n* = 20 cells examined over 3 biologically independent samples, mean ± SD, *p* < 0.0001 one-way ANOVA test) **d** Nb-C6 could activate SARM1 in vitro, measured by reverse cycling assay. The cleaned cell lysate of HEK293 overexperssing dtSARM1, pre-incubated with 10 nM Nb-C6, or Nb-1053 as a negative control, on ice for 1 h, was added to the reaction system containing the substrates, 10 μM cADPR and 100 μM nicotinamide, with or without 100 μM NMN, for the indicated time. The produced NAD was measured by the cycling assay. (*n* = 4 biological independent experiments, mean ± SD) **e** The reaction initial rates in (d) were normalized with that of the sample, NMN-activated SARM1, and plotted into bar graph. (*n* = 4 biological independent experiments, mean ± SD) **f**, **g** Nb-C6 elevated the cellular cADPR levels. The HEK293T cells (**f**) or HEK293 cells carrying the inducible SARM1 expression cassette (**g**) were transiently transfected with the plasmids encoding the YFP-fusion nanobody, C6 or 1053. The cells were harvested 48 h post-transfection and the cellular cADPR contents were measured by cycling assay and proteins by western blots (Supplementary Fig. 2e, f) (*n* = 3 biologically independent experiments, mean ± SD, *p* < 0.0001 one-way ANOVA test). Source data are provided as a Source Data file.

We used surface plasmon resonance (SPR) to confirm that Nb-C6 indeed targets only the activated form of SARM1. As described in the Methods section, purified recombinant SARM1 with a strep-tag was first captured on the surface of an SPR chip coated with Strep-Tactin^TM^ XT proteins. The two-state kinetics model provided an affinity (*K*_D_) of ~25 nM as measured for the binding of Nb-C6 to the activated complex SARM1^NMN^ (abbreviation: P^M^ means the protein or domain “P” loaded with the small molecule “M” in the whole text) (Supplementary Fig. 1). The effect of varying the NNM/NAD ratio was assessed next. A running protocol was designed to gradually shift the binding ligand from NAD to NMN and measure the resultant changes in Nb-C6 binding to immobilized SARM1 (Fig. 2a, right panel). As shown in Supplementary Fig. 2a, at a high NAD concentration (1.6 mM), the binding of Nb-C6 to SARM1 was very low. As the ligand was gradually shifted to NMN, the binding proportionally increased to a higher level at 100 µM of NMN. We plotted the Nb-C6 binding as a function of the molar ratio of NAD and NMN, which was fitted into a linear line (equation in Fig. 2a, left) with *R*^2^ = 0.97, indicating that the NAD/NMN ratio determines the Nb-C6 binding on and the activation of SARM1

We next tested whether Nb-C6 can detect the activated form of SARM1 in cells. Nb-C6 fused with a fluorescent tag, mNeonGreen (mNG), was produced and used for visualizing the activated SARM1. HEK293 cells exogenously express low levels of SARM1 that can be activated by the permeant NMN mimetic CZ-48^8^. Positive staining by Nb-C6 was seen in the cells only after CZ-48 treatment but not in the untreated control cells (C6-mNG, Fig. 2b, green signals). For comparison, we generated polyclonal anti-SARM1, which was raised against the recombinant SARM1 and could recognize SARM1 in both conditions (αSARM1, Fig. 2b, red signals). The Nb-C6 signals were perfectly colocalized with the anti-SARM1 (Supplementary Fig. 2b, c) staining, with the coefficient values (PC, M1, and M2)^27^ close to 1. It is believed that SARM1, with its N-terminal localization signal is normally expressed at the outer mitochondrial membrane^8,19^. This was further confirmed here, as both the Nb-C6 and anti-SARM1 signals were colocalized with anti-Tom20 (mitochondrial marker; Fig. 2b, yellow signals; Supplementary Fig. 2d, e). The Nb-C6 signals were then quantified by normalizing pixel-by-pixel to the anti-SARM1 signal, which represented the total amounts of SARM1 in the same cell (ratio, Fig. 2b, last panels). The average of the ratios from approximately 20 cells (Fig. 2c) demonstrated a significant increase in Nb-C6 binding upon CZ-48-induced activation of SARM1. The results confirm the conformational specificity of Nb-C6 and suggest that the activation of mitochondria-localized SARM1 by NMN in live cells also undergoes conformational changes and exposes the recognition site for Nb-C6.

We reasoned that Nb-C6 itself may be able to promote the activation of SARM1, since its binding site on SARM1 could be exposed randomly at low levels even without activation. The presence of Nb-C6 could then bind to and stabilize the active form. This is the case as shown in Fig. 2d–g. When preincubated with Nb-C6 in vitro, SARM1 can be activated but much less significantly compared with those activated with NMN and both (Fig. 2d; quantification is in **2e**), which was consistent with the expectation. Nb-C6, with a soluble tag YFP (C6-YFP), was then transfected and overexpressed in two cell types. HEK293T cells endogenously express significant levels of SARM1, which can be activated by CZ-48 treatment^8^, and, when overexpressing C6-YFP (Supplementary Fig. 2f), showed elevated cADPR levels, indicating that Nb-C6 alone can activate endogenous SARM1 (Fig. 2f, C6-YFP). No cADPR increase was observed in the control cells overexpressing an irrelevant Nb, i.e., 1053-YFP^26^. In another cell line, HEK293 cells expressing inducible SARM1 also showed higher levels of cADPR after transfection with Nb-C6 as compared to transfection with 1053-YFP (Fig. 2g and Supplementary Fig. 2g).

The above results fully validate that Nb-C6 recognizes only the activated form of SARM1 and minimally recognizes the inactive form and show that Nb-C6 is a valuable tool for activating endogenous and exogenous SARM1 in live cells.

### Overall cryo-EM structure of the NMN-loaded SARM1 complexed with Nb-C6

We next determined the stabilized structure of NMN-treated SARM1 by cryo-EM. Recombinant SARM1, with the N-terminal 27-aa segment replaced with a Flag/Strep tag, was prepared and characterized as described previously^23^. The recombinant SARM1 was preincubated with NMN and Nb-C6.

**Fig. 3:**
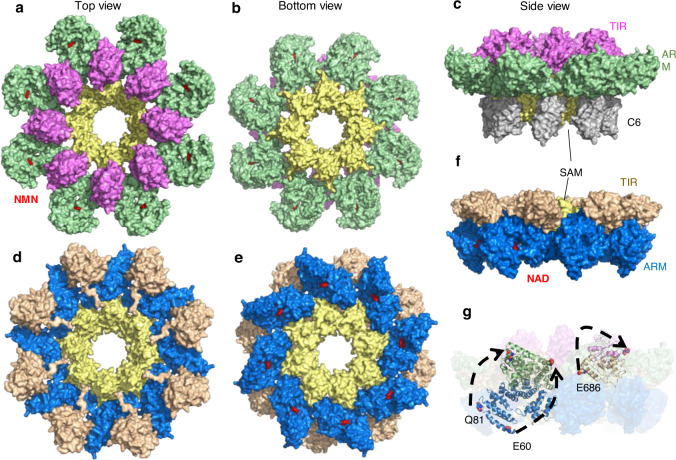
Overall structures of active or inactive SARM1. **a**–**c** Structure of the SARM1^NMN^/Nb-C6 complex. The order of addition in forming the complex is SARM1 first, NMN second, and then Nb-C6. **a** top view; **b** bottom view; **c** side view. SARM1 and Nb-C6 are shown as surface models with ARM domain colored in green, TIR domain in magenta, SAM domain in yellow and Nb-C6 in grey. NMN is shown as sphere in red. Each Nb-C6 binds one SARM1 protomer. **d**–**f** Overall structure of SARM1^NAD^ (PDB 7ANW). **d** top view; **e** bottom view; **f** side view. SARM1 is shown as surface model with ARM domain colored in blue, TIR domain in wheat and SAM domain in yellow. NAD is shown as sphere model in red. **g** The side view of superposition of NMN- and NAD-bound SARM1. For clarity, only one protomer in NMN-bound and NAD-bound forms are shown as cartoon models and other protomers are shown as surface, and Nb-C6 in SARM1^NMN^ complex was hidden. SARM1^NMN^ is colored as in panels (**a**–**c**) and SARM1^NAD^ is colored as in panels (**d**–**f**). Residues E60 and Q81 in ARM domain and E686 in TIR domain are shown as spheres. The re-orientation of corresponding residues is indicated by black arrows.

Cryo-EM images were collected and processed as detailed in the Methods section and Supplementary Fig. 3. With an overall resolution of approximately 2.7 Å, the structure of the SARM1^NMN^/Nb-C6 complex displays two layers of octameric rings, containing 16 SARM1 protomers and 16 Nb-C6 molecules (Supplementary Fig. 4a). Nb-C6 bound mainly to the SAM domain and dimerized at the C-terminus, stabilizing the whole structure like pillars (Fig. 3c and Supplementary Fig. 4a). The two octamers in the structure were almost identical; hence, we analyzed only one octamer.

The TIR domains were less stable, as determined by particle imaging analyses and the most flexible part was the outer ring formed by the ARM domains (Supplementary Fig. 4b). Further focused 3D classification around the ARM and TIR domains was performed on symmetry-expanded datasets, generating three major conformations, named Class 1, Class 2 and Class 3 (Supplementary Figs. 3c and 4c–e). Class 1 and Class 2, with clear densities around ARM1-4 motifs (Supplementary Fig. 4d, e), were further processed by multibody refinement^28^, which generated focused density maps. The final resolution of Class 1 and Class 2 was 3.7 Å and 3.8 Å, respectively; while the local resolution was 3.6-4.2 Å for Class 1 or 3.6-4.5 Å for Class 2 (Supplementary Fig. 5a, b). The ARM-TIR domains in Class 2 rotated approximately 5° clockwise compared with those in Class 1 as revealed via the superposition of the SAM domains (Supplementary Fig. 6a, b), but the ARM domains in both classes were almost identical, with a root mean square deviation (RMSD) value of 1.06 for all main-chain atoms of V61-E400 (Supplementary Fig. 6c), indicating that the ARM-TIR domains moved as a whole. Using the structural information obtained from crystallography of individual domains, all the residues in SARM1 could be traced except those in the linker between the SAM and TIR domains.

The SARM1 octamer complexed with NMN, i.e., SARM1^NMN^, was assembled through the oligomerization of the SAM domains (Fig. 3a-c, yellow), which was almost identical to that for the previously published SARM1^NAD^ (PDB 7ANW; Fig. 3d–f, yellow) with an RMSD of ~0.62 Å for all Cα atoms in the SAM domain. The overall architecture of SARM1^NMN^ exhibited a “blooming lotus” shape (Fig. 3a–c), in sharp contrast to the compact “doughnut” structure of SARM1^NAD^ (Fig. 3d–f). The ARM-TIR domains, as a whole, swung out from the SAM domain and rotated ~139°, with 73 Å for the N-terminal residue E60, as compared to that in SARM1^NAD^ (Fig. 3g, blue to green for the ARM domain; wheat to magenta for the TIR domain). This large conformational change of SARM1 following NMN binding exposed regions recognized by Nb-C6, accounting for its specificity only for NMN-activated SARM1.

During the submission of this work, Shi et al. published the cryo-EM structure of the SARM1^NMN^ complex, although only the SAM and ARM domains could be resolved (PDB 7NAL). In this ARM-SAM^NMN^ assembly, the ARM domain also swings upward with respect to SARM1^NAD^ (PDB 7ANW), but the rotation angle and movement distance are different from those of SARM1^NMN^/Nb-C6 (Supplementary Fig. 7a), representing alternative states during SARM1 activation.

### Induction of significant structural changes in the ARM domain by NMN

**Fig. 4:**
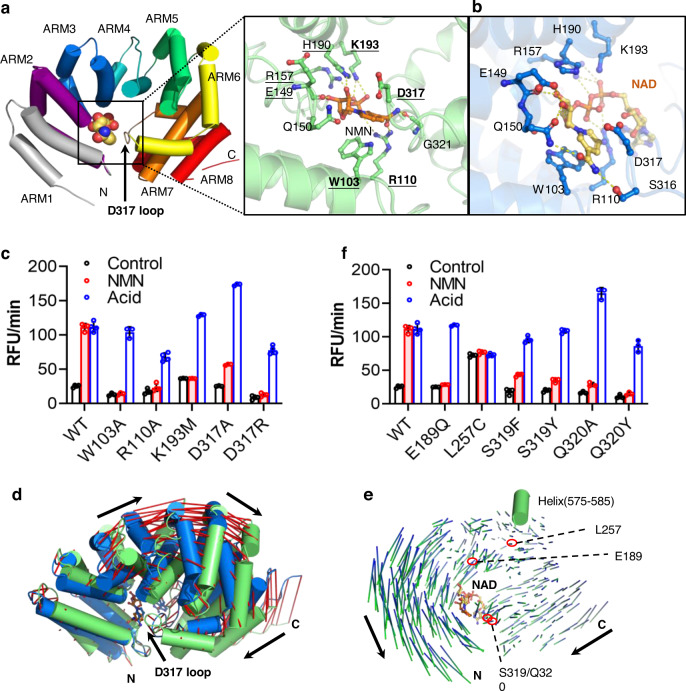
Conformational changes of the ARM domain induced upon NMN binding. **a** The structure of ARM^NMN^. ARM domain is shown as cartoon, with separate armadillo motifs in different colors, and NMN molecule is shown as sphere. The D317 loop is indicated with a black arrow. The right panel shows the zoom-in view of the NMN-binding pocket. NMN and the NMN-interacting residues are shown as ball-and-stick models, with NMN colored with gold carbon atoms and amino acids with green carbon atoms. Hydrogen bonds are shown as yellow dashed lines. The residues mutated in this work are labeled in bold and the reported mutations with underlines. **b** The binding pocket of NAD in ARM domain. SARM1^NAD^ (PDB 7ANW) is superimposed onto SARM1^NMN^ at the same orientation of panel a. Amino acids are shown with blue carbon atoms and NAD with yellow carbon atoms. **c**, **f** The constitutive activity and NMN-responsiveness of the SARM1 mutants. HEK293 cells carrying the inducible expression cassette for the mutants were treated with 1 μg/mL doxycycline for 24 h, the proteins were extracted with PBS containing 4 mM Digitonin and were applied to PC6 assay following the treatment of 100 μM NMN or 0.1 M NaOAc, pH 4.0^23^. c Mutation of the residues directly interacting with NMN; f. Mutation of the residues involved in a conformational change of ARM. (*n* ≥ 3 biological independent experiments, mean ± SD) **d**, **e** Conformational changes of ARM domain in SARM1^NMN^ (green) compared to SARM1^NAD^ (PDB 7ANW) (blue). Residues 93-104 are aligned in panel (d) and positional shifts of Cα atoms are indicated by red lines and black arrows. The inward shift of the D317 loop is indicated with a black arrow. In panel (**e**), residues 575–585 in the TIR domain (green cylinder) are superimposed and the positional shifts of Cα atoms in the ARM domain are indicated by blue-green lines and black arrows. Source data are provided as a Source Data file.

NMN bound to the ARM domain, a compact upside down ω-shaped structure formed by eight ARM repeats, each composed of α helices. The binding site was a pocket enclosed by the H3 helix from ARM1, the H1 helix from ARM2 and the loop connecting ARM6-7 (hereafter named the D317 loop) (Fig. 4a). The NMN binding modes in the two conformational classes of ARM domains are almost identical (Supplementary Fig. 7c) and only the mode in Class 2 will be discussed due to its clearer density and better model-map fit. NMN directly interacted with seven residues including W103, R110, E149, Q150, R157, H190, K193, S316, and G321 (Fig. 4a, right panel). The pyridine ring of NMN packed against the indole ring of residue W103, forming a parallel π-π stack, while the amide group formed hydrogen bonds with the main-chain carboxyl group of G321 (Fig. 4a, right panel). The ribose part of NMN also formed hydrogen bonds with the main-chain carboxyl group of residue E149. The phosphate group formed salt-bridges with the side chains of R110, R157, and K193.

The NMN-binding site, in fact, was the same as that for NAD as observed in inactive SARM1^NAD^ (Fig. 4b), accounting for the two ligands being competitive^11^. However, the nicotinamide group in NAD was oriented upward while that in NMN was oriented downward (Fig. 4a, b). The amide moiety in the nicotinamide group of NAD formed an H-bond with the side-chain hydroxyl of residue S316 instead of the main-chain carboxyl of residue G321 in ARM^NMN^. The additional phosphate, ribose, and adenosine groups in NAD formed interactions with the D317 loop and pushed it outward ~5 Å. The NMN binding mode observed in this structure is almost identical to that in the newly published SARM1 (PDB 7NAL) with an RMSD value of 0.93 Å for the main chain atoms of V61-E400 (Supplementary Fig. 7b). The structural differences between ARM^NMN^ and ARM^NAD^ can account for NMN being an activator and NAD being an inhibitor.

Mutational studies confirm the functional importance of the NMN/NAD binding site. HEK293 cells were transfected and expressed SARM1 with mutations in residues, W103, R110, K193, and D317 (bold in Fig. 4a) and were tested for the enzymatic activity and responsiveness to NMN activation, using our recently developed protocols^23^. As expected, all the mutants became unresponsive to NMN (Fig. 4c, black bars vs. white bars; the measurement of protein expression is in Supplementary Fig. 8a), while all could still be activated by acid (Fig. 4c, gray bars). We have recently shown that acid activates SARM1 by breaking salt bridges between ARM and TIR, resulting in full expression of its enzymatic activities^23^. The results support that these residues are critical to NMN binding. This finding is also consistent with the previous results showing that similar mutations (underlined in Fig. 4a) can eliminate the responsiveness to nicotinamide ribose-induced NMN accumulation in neurons^11^.

Next, we investigated the NMN-induced conformational change of the ARM domain by aligning ARM^NMN^ (Fig. 4d, e, green) and ARM^NAD^ (blue). Large conformational changes were clearly visible after aligning the H3 helix of ARM1, residues 93-104, as indicated by the red-line shift in Fig. 4d. ARM^NMN^ showed significant inward bending compared to ARM^NAD^, with the C-terminal ARM7-8 motifs moving much closer toward the N-terminus of the ARM domain, pushing the D317 loop to insert deeper into the NMN binding pocket. Similar large changes were seen by superimposing residues 575-585 in the ARM:TIR interface (Fig. 4e, green cylinder). The length of the lines in Fig. 4e indicates the movement distance of individual residues, while the color blue (ARM^NAD^) to green (ARM^NMN^) indicates the inward direction of the movement. The large conformational change is also evidenced by the large RMSD (~2.4 Å) yielded from the superposition between ARM^NMN^ and ARM^NAD^ of hSARM1, which is mainly attributed to the shift of helices on the convex side and also the inward movement of ARM7-8 motifs (Fig. 4d).

It is likely that the inward bending of the ARM domain induced by NMN is necessary for the enzymatic activation of SARM1. To test this hypothesis, we mutated several residues that could potentially contribute to the bending, including E189, L257, S319, and Q320 (locations indicated in Fig. 4e). The choice of these residues was suggested by structural modeling, indicating that replacing S319 or Q320 with a tyrosine could elicit strong hydrophobic interactions with residues in the H3 helix of ARM1 and the H1 helix of ARM2 and hence could interfere with the inward movement of the D317 loop upon NMN binding (Supplementary Fig. 8b). Consistently, the enzymatic activity of the mutants showed that NMN-induced activation was abolished in most of these mutants, as shown in Fig. 4f and Supplementary Fig. 8c. An exception was L257C, which was constitutively active, with its basal activity already being similar to that activated by NMN or acid. The structural modeling in Supplementary Fig. 8d indicates that the cysteine side chain of L257C could reduce the hydrophobic interaction and might form a disulfide bond with C215 as well, promoting the inward bending of ARM4-5 and rendering the mutant constitutively active. These results support that the inward bending of the ARM domain is necessary for NMN-induced SARM1 activation.

### NMN-induced release of the ARM domain from SAM

**Fig. 5:**
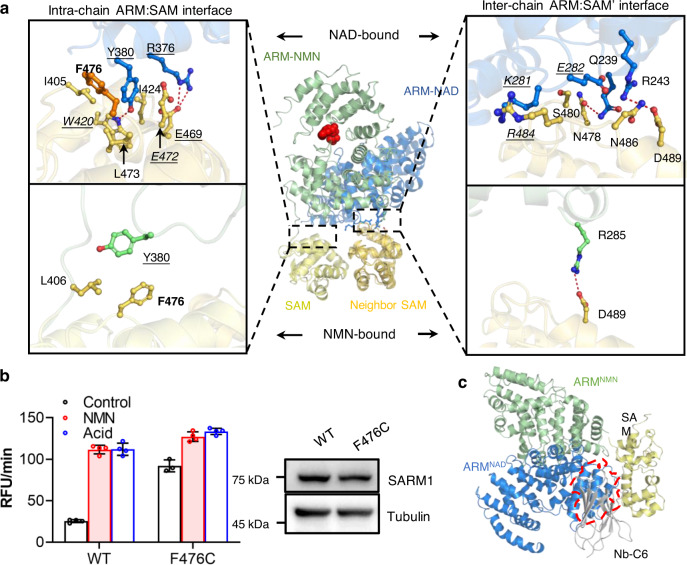
Releasing ARM from SAM domain allows Nb-C6 binding. **a** Superposition of SARM1^NMN^ onto SARM1^NAD^ (PDB 7CM6) via the SAM domains. Left panels: intra-chain ARM-SAM interfaces; Right panels: inter-chain ARM-SAM’ interfaces; Upper panels: NAD-bound; lower panels: NMN-bound. The ARM and SAM domains are shown in green and gold in SARM1^NMN^, respectively; and they are colored in blue and orange in SARM1^NAD^, respectively. Key interaction residues at the interfaces are shown in ball-and-stick models and labeled. The H-bonds and salt-bridge are shown as red dash lines. The residues mutated in this work are labeled in bold and the reported mutations with underlines; the ones behave similar to wild-type labeled in italic. **b** The activity and NMN-responsiveness of the SARM1 mutants measured as Fig. 4c. (*n* ≥ 3 biological independent experiments, mean ± SD) **c** Superposition of SARM1^NMN^ onto SARM1^NAD^ (PDB 7CM6) via the SAM domains. The proteins are shown as cartoon models with SAM domain in yellow, nanobody in grey and ARM domain of SARM1^NAD^ and SARM1^NMN^ in blue and green, respectively. The overlapping area was highlighted by a red dash line. Source data are provided as a Source Data file.

Next, we investigated how NMN-induced bending of ARM leads to its dissociation from the SAM domain. The ARM domain of each protomer interacts with both its own SAM (intrachain) and the adjacent SAM (interchain) as well. Figure 5a shows the involved interfaces. When complexed with NAD, two key residues in ARM were critical for the intrachain ARM:SAM association (Fig. 5a, upper left panels). Residue R376 formed salt-bridges with E469 in SAM, while Y380 was inserted into a hydrophobic pocket formed by residues I405, W420, L424, L473, and F476. In particular, the π-π stack between Y380 and F476 might contribute to maintaining the ARM-SAM association. Mutating F476 to cysteine abolished the stacking and weakened the association, rendering the mutant constitutively active (Fig. 5b). Likewise, the mutants R376A, E469A, and Y380A (underlined in Fig. 5a) also had increased basal activities^29^, supporting that the intrachain ARM:SAM interactions are critical for maintaining the autoinhibitory state. In the interchain ARM:SAM’ interface (Fig. 5a, upper right panel), several H-bonds and salt bridges were observed. These interchain associations, however, did not seem to contribute to autoinhibition because all the tested mutations (italic in Fig. 5a, upper right panel) behaved like the wild type^29^.

When complexed with NMN, both the intra- and interchain ARM-SAM associations were diminished, with Y380 flipping away from F476 in the intrachain SAM and only one salt bridge (R285-D489) remaining in the interchain interface (Fig. 5a, lower panel). The binding of NMN to ARM induced large conformational changes in the domain, comprising an inward shift of ARM7-8 repeats toward the NMN-binding pocket and the rotation of the whole ARM domain, exposing binding sites for Nb-C6 (Supplementary Fig. 9a). As a result, the distances increased between all the interacting residues observed in the SARM1^NAD^ complex, rendering their interactions ineffective. The diminished interactions release SAM from ARM and expose the site for Nb-C6 to bind. The nanobody mainly bound to the SAM domain with a lesser extent to the ARM-SAM linker (Supplementary Fig. 9b). The binding interface between Nb-C6 and the SAM domain was largely overlapped with the intrachain ARM:SAM interface (Fig. 5c), accounting for the fact that Nb-C6 does not recognize inactive SARM1 as the binding site is obscured by ARM.

### Induction of conformational changes in the TIR domains by NMN

Upon binding of Nb-C6 and NMN to SARM1, the ARM and TIR domains swung out as a whole with minimal changes in the interface between them (Supplementary Fig. 10a). The movement and the accompanying bending of the ARM domain described above, however, did result in shifting away of the R216 residue in the intrachain ARM domain (Supplementary Fig. 10a, black arrow) from E689 in the TIR domain, breaking the salt bridge between them. The linkage was believed to also be essential for its autoinhibition^23,29^.

Superimposing TIR from SARM1^NMN^ with that from SARM1^NAD^ showed that the overall structures were almost identical, including the BB loop (Supplementary Fig. 10b, magenta/blue loops), which is considered to be important in determining the enzymatic activity state^6,30^. The conformation of the BB loop in both complexes appeared to be constrained by the linker between SAM and TIR (Supplementary Fig. 10b, in sphere mode) and might hinder the entrance of the substrate into the catalytic pocket. Upon activation, TIR domains leave ARM domains, and the constraint on the BB loop would be relieved allowing the entrance of substrates, as evidenced in the structures of the TIR domain in the complex with substrate mimetics 1AD, 2AD, 3AD, and ara-F-ADPR (PDB 7NAG, 7NAH, 7NAI, and 7NAJ) (Supplementary Fig. 10c). The relief of the constraint was also observed by HDX-MS and XL-MS experiments described below.

**Fig. 6:**
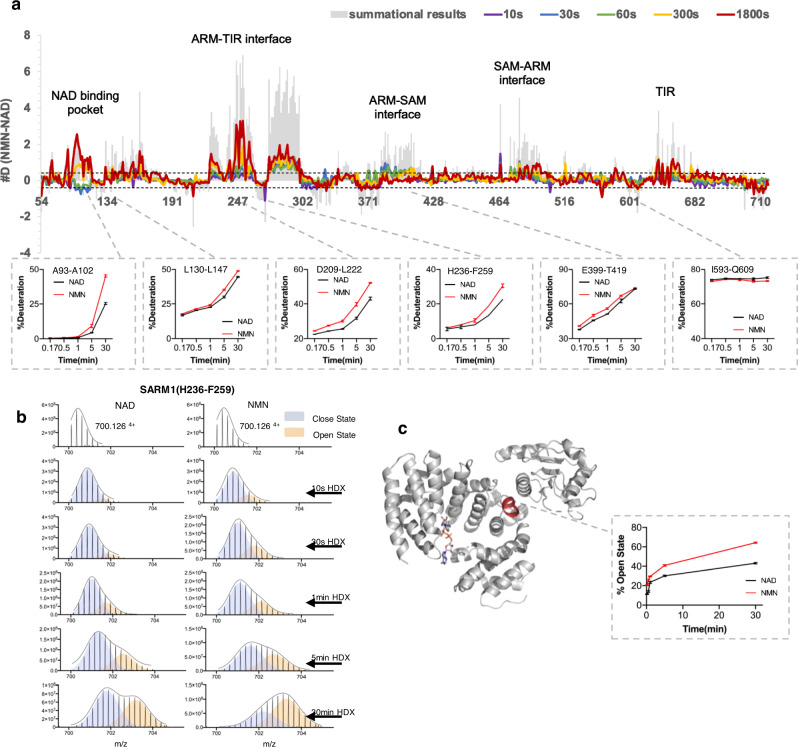
HDX-MS reveals the conformational dynamics of NMN- and NAD-bound SARM1 octamers in solution. **a** Plot representing differences in deuterium uptake between NMN- and NAD-bound SARM1, with each data point representing the central residue of an individual peptide (ordered from N- to C- terminus). The Y-axis represents the total difference in the number of deuterium uptake for a given peptide, between NMN and NAD bound SARM1 at each time point. Positive bars indicate an increase in the exchange of the corresponding peptide in the presence of NMN and reveal that several distinct regions are affected by ligand binding. Horizontal dotted lines represent the threshold above which deuterium uptake differences were considered significant (>0.4 Da). HDX data statistics is given in Supplementary Table 1, and details are in Supplementary Data 1. The Time course of HDX incorporation in the presence of NAD or NMN for several representative peptides are shown. **b** Bimodal isotopic envelopes for the SARM1 helix α13 (peptide spanning residues 236–259). Relative amount in the closed and open state in the presence of NAD and NMN as seen by HDX. Deuteration time points are indicated. Open-to-close transition kinetics for SARM1 helix α13 is calculated by a fit of two gaussians. The fitted peak area parameter was used to calculate the relative amount of open state by taking the ratio of high and the sum of the high and low mass subpopulations. **c** Structure of the NAD bound SARM1 showing ARM-TIR domain interface with the ARM helix α13 indicated in red. NAD ligand is shown as a stick model. All of the experiments were technically replicated three times (*n* = 3 independent experiments, mean ± SD). Source data are provided as a Source Data file.

SARM1 was preincubated with the excess of either NAD or NMN and followed by HDX labeling for various periods, starting with 10 s, which is sufficient to deuterate fully solvated amides^31^. The coverage map of SARM1 was composed of 557 peptides spanning ~94% percent of the exchangeable amides (Supplementary Table 1 and Supplementary Data 1). The HDX data are consistent with the secondary structure of ARM, SAM and TIR domains, with alpha helices incorporating less deuterium to a lower degree than loop regions or less well-defined helices (Supplementary Fig. 11). While the majority of the peptides followed EX2 type HDX kinetics, the peptide isotopic peaks corresponding to helix α13 (exemplified by peptide spanning residues H236-F259) in the ARM domain formed a clear bimodal distribution. A bimodal distribution could result from EX1-type HDX kinetics, in which two distinct protein conformations with different solvent accessibility interconvert during the HDX labeling period^32,33^. The hydrogen exchange observed in these peptides can be described by the EX1 kinetic regime, where the intrinsic hydrogen exchange rate is much higher than the refolding rate. In EX1 kinetics regime, all the amide hydrogens can exchange with deuterium during one unfolding event, resulting in two distinct mass envelopes. The lower mass envelope represents the less accessible closed state (Fig. 6b, blue shade), and the higher mass envelope represents the more accessible open state (Fig. 6b, yellow shade). The two molecular mass envelopes were localized to the ARM-TIR interaction interface of the ARM domain (helix α13) (Fig. 6c). This behavior was evident at 10 s to 30 min of exchange. In SARM1, the less accessible molecular mass envelope gradually converted into a more accessible state (Fig. 6b, c). Importantly, preincubation with NAD significantly stabilized the less accessible state and slowed down the conversion to more accessible state when compared to SARM1^NMN^ (Fig. 6b, c). ARM helix α13 is responsible for the direct interaction and recruitment of the TIR domain to the periphery of the SARM1 homo-octameric ring. Furthermore, the NAD-bound state showed a reduction in deuteration levels in the peptide spanning residues at the ARM-SAM and ARM-TIR interaction interfaces as well as the NAD binding pocket in ARM domain (Fig. 6a). These changes were consistent with a major stabilization effect on the SARM1 dynamics when NAD was present, and support the model where the binding of NAD stabilizes the interaction between TIR, ARM and SAM domains, resulting in the “donut-shape”, two-ring compact conformation. In contrast, the SARM1^NMN^ complex formed a more dynamic structure, consistent with the TIR-ARM domain dissociation resulting in a faster conversion of helix α13 to the open state.

We next used crosslinking–mass spectrometry (XL-MS) to compare the conformational differences between SARM1 complexed with NMN and NAD. We used the MS-cleavable crosslinker DSBU (disuccinimidyl dibutyric urea), which connects primary amine groups of lysine residues within Cα-Cα distances up to ~30 Å^34^. Three different SARM1 concentrations (1.25 µM, 2.5 µM, and 5 µM) were used, at which the protein forms multimers in solution (Supplementary Fig. 12). SARM1 was preincubated with saturating concentrations of NAD or NMN prior to crosslinking, followed by LC-MS identification (Supplementary Data 2). We then used the information derived from XL-MS and cryo-EM for modeling SARM1 regions of interaction, based on a maximum 30 Å Cα–Cα distance constraint between two crosslinked lysine residues. Since SARM1 forms multimers, the XL-MS approach used in this study is not capable of differentiating intramolecular from intermolecular links. Interpretation of the links as intra- or intermolecular was therefore aided by high-resolution cryo-EM structures.

For SARM1 complexed with NAD, 87% of the intra- and intermolecular XLs identified were consistent with the domain structures and complex architecture observed in the cryo-EM structure of SARM1^NAD^ (PDB 7CM6) (Supplementary Fig. 13a and 14). The other 13% were unsatisfactory XLs of residues with distances >30 Å or steric clashes, indicating the presence of alternate domain conformations in the solution. These results are consistent with a previous study where ~80% of the particles were found in the two-ring, compact conformation when incubated with NAD, versus partially or completely dissembled particles where only the inner ring is visible^10^. The removal of NAD and incubation with NMN were expected to trigger the disassembly of the compact conformation by ARM domain rearrangements and the dimerization of TIR domains, which enables the enzymatic activities of TIR. Indeed, a 20% increase in the number of unsatisfactory XLs was observed when XLs from the SARM1^NMN^ sample were mapped onto the Nb-C6 complex structure obtained in this study or the NAD-bound compact structure (PDB 7CM6) (Supplementary Fig. 14). NMN-specific XLs were mapped to the ARM and TIR domains, and ~60% were unsatisfactory when mapped onto the Nb-C6 stabilized structure (Supplementary Fig. 13b–d), indicating alternative orientations of ARM and TIR domains when in solution. Furthermore, XLs of residues K636-K694 and K602-K694 involve lysine residues in regions of defined secondary structures located on opposite faces of the TIR domain. These residues were unlikely to form intramolecular XLs since the vectors for these XLs passed directly through the TIR domain. We believe these XLs arose as a consequence of intermolecular TIR-TIR domain interactions. We mapped these XLs onto the newly published cryo-EM structure of a TIR multimer (PDB 7NAK)^24^. The distances of all lysine pairs were within 30 Å in the dimer with the BB loop as the interface, with no steric hindrance (Supplementary Fig. 13e), indicating that this might be the predominant interaction in the solution.

**Fig. 7:**
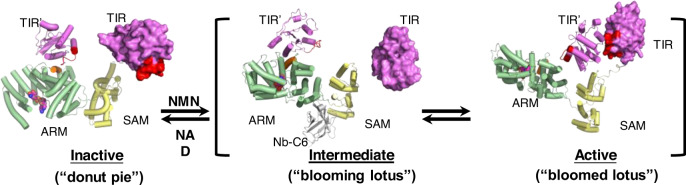
A proposed model for the transition between the inactive and active states of SARM1. SARM1 was shown with the ARM domain in green, SAM domain in yellow, and TIR domain in magenta. TIR’ is the domain from a neighboring SARM1 protomer. The 13 helix (residues 249-259) in the ARM domain was colored in orange. BB loop in the TIR domain was colored in red.

Taken together, the HDX-MS and XL-MS data suggest that the NMN-activated SARM1 undergoes a series of ARM and TIR domain rearrangements in solution. The Nb-C6-stabilized structure thus may represent an intermediate toward this final state of activation. As depicted in Fig. 7, in this final state, the TIR domain disengages from ARM and directly interacts with the neighboring TIR, forming multimers and resulting in enzymatic activities. This conformational change is coupled with ARM domain rearrangements, contributing to the disassembly of the peripheral ring of SARM1.

## Discussion

SARM1 has attracted substantial interest because of its role in AxD^4^. The finding that it is an NADase^7^ has led to the proposal that its activation after axonal injury can deplete cellular NAD and cause AxD. Recent results show that SARM1 is not just a degradative NADase but also an autoregulated signaling enzyme responsible for producing two calcium mobilizing messengers, cADPR and NAADP^8,23^. For example, in paclitaxel-induced peripheral neuropathy, SARM1 is activated to produce cADPR, resulting in the increase of calcium and AxD^17^. We and others have shown that the enzyme activities of SARM1 are specifically activated by NMN^8,11^. Understanding the mechanism of its activation is clearly important and a structural approach is the most direct.

Combining the results obtained using cryo-EM and crystallography of various domains has produced a high-resolution structure of the octameric SARM1 in the inactive state^9–11,22,23^. The flexibility of the active SARM1 hindered the resolution of its structure, which has been overcome recently with the help of an NAD derivative, 1AD, acting as a “molecular glue” and stabilizing the TIR oligomers^24^. We approached this problem by generating a conformation-specific nanobody, Nb-C6 that binds only to and stabilizes the activated SARM1, allowing the determination of a full-length structure by cryo-EM.

Four lines of evidence support that Nb-C6 is specific to the activated SARM1 in solution and cells. First, Nb-C6 immunoprecipitated SARM1 only when it was activated by NMN (Fig. 1). Second, SPR documents that Nb-C6 has a much higher preference for SARM1^NMN^ over SARM1^NAD^ or ligand-free SARM1 (Fig. 2a and Fig. S2a). Third, immunofluorescence staining by Nb-C6 was observed only when cells were activated by the permeant NMN mimetic, CZ-48 (Fig. 2). Finally, Nb-C6 can activate the endogenous SARM1 in cells to produce cADPR (Fig. 2f, g), although to a lesser extent than CZ-48, a permeant NMN analog.

In our cryo-EM structure, SARM1 is also an octamer assembled through the SAM domains. In contrast to the donut shape of the inactive SARM1 complexed with NAD (Fig. 3d–f; Fig. 7, left panel), the NMN-activated and Nb-stabilized form resembles a “blooming lotus” shape, with ARM and TIR extended out to form the “petals” (Fig. 3a–c; Fig. 7, middle panel). The nanobody binds mainly to the SAM and partially with the ARM domains, essentially covering the hinge between the SAM and ARM and stabilizing the whole structure. The NMN-loaded ARM domain exhibits a significant inward bending as compared to ARM^NAD^. The NMN-induced conformational change of ARM leads to its release from SAM, which exposes the binding site of Nb-C6. The TIR domains swing out together with and still sit upon ARM domains and remain separated from the neighboring TIR, indicating that the structure represents an intermediate state of SARM1 activation.

Proceeding to the final active state, the TIR is released from ARM and can be chemically crosslinked to its neighboring TIR of the active octamer, as we showed using XL-MS (Supplementary Fig. 13e). This is consistent with the results showing that isolated TIR domains must dimerize before they become enzymatically active^6^ and the recently reported structure of the TIR domain oligomer in the SARM1 activated state.

The interface of the TIR dimers is mainly composed of weak interactions. We speculate that in the absence of NMN, or when it is metabolized, the TIR domains in the dimers might revert to complexing with ARM in the intermediate state, and SARM1 re-enter the inactive state.

The limitations of crystallography and cryo-EM for structural determination are well known. Packing protein molecules in a crystal can introduce distortions. Cryo-EM has fewer packing changes but is still limited by the flexibility of the molecules and high-resolution reconstruction from a selected subset of particles. In this study we used HDX- and XL-MS to assess the dynamics of the fully active SARM1 in solution. This combination of approaches has provided a better understanding of the activation mechanism of this novel autoinhibited signaling molecule. Our data imply that the homodimerization or oligomerization of TIR domains in activated SARM1 but substrate-free SARM1 should be transient or unstable but can be captured and stabilized by the compound 1AD^24^.

In summary, Nb-C6 not only provides an important understanding of the structural changes during the activation process but also allows imaging and localization of the active SARM1 in cells. Nb-C6 thus supplements well with the permeant probe, PC6, which we previously developed for visualizing SARM1 activity in live cells^23^. Furthermore, transfection and expression of Nb-C6 can activate endogenous SARM1, providing a means to manipulate SARM1 in live cells in addition to the permeant activator CZ-48 we have synthesized^8^.

## Methods

### Bacteria and cell lines

OverExpress^TM^ *E. coli* C43(DE3) obtained from Weidi Biotechnology (Shanghai) was used to express the recombinant proteins.

Expi293F cells, obtained from Thermo Fisher (A14527), were cultured in serum-free SMM-293TII media (Sino Biological M293TII) at 37 °C in a humidified, 5% CO_2_ atmosphere incubator, shaking at 150 rpm. HEK293 (ATCC CRL-1573) and HEK293T (ATCC CRL-3216) cells obtained from ATCC were cultured at 37 °C with 5% CO_2_ in DMEM supplemented with 10% FBS and 1% penicillin/streptomycin.

### Reagents

NAD, NMN, Digitonin, Poly-L-lysine, KH_2_PO_4_, NH_4_HCO_3_, chloroacetamide, polyethylenimine (PEI), and urea were purchased from Sigma-Aldrich. DMEM, Trypsin-EDTA, penicillin/streptomycin solution, Lipofectamine 2000, formic acid, and acetonitrile were purchased from Thermo Fisher. FBS was obtained from PAN Biotech. SMM-293TII media was obtained from Sino Biological. Ni-Excel column, HiTrap Q column, CM5 sensor were obtained from Cytiva. Strep-Tactin resin, StrepTactin^TM^ XT SPR kit were obtained from IBA. General chemicals were purchased from Aladdin, Macklin, or Sangon Biotech (Shanghai).

### Immunization and screening for Nanobodies (Nbs)

The immunization and library construction were conducted in Shenzhen KangTi Life Technology Co., Ltd (AlpaLife). The study involving alpaca raising or immunizations at Shenzhen KangTi Life Technology Co., Ltd (AlpaLife) complies with the relevant ethical regulations for animal testing and research. Briefly, a healthy, adult, male alpaca was immunized with the recombinant dN-SARM1, full-length SARM1 with the N-terminal 27-aa segment replaced by a BC2T-peptide, which was expressed in HEK293T cells^8^ and immunoprecipitated by BC2Nb-conjugated beads^25^. The antigen on beads, mixed with Freund’s adjuvant, was injected subcutaneously for 6 times during 3 months. A VHH phagemid library was constructed from the mRNAs of the immunized peripheral lymphocytes, with a titer around 2.5 × 10^8^. Library screening was performed by the routine protocol of phage display, panning and colony ELISA^35^. Briefly, *E.coli* TG1 transformed with the phagemid library was grown at 250 rpm, 37 °C to *OD*_600_ 0.4-0.5 and then cocultured overnight with the helper phages, allowing packaging into the recombinant phage particles, which were then precipitated with PEG/NaCl and resuspended with PBS. Biopanning was performed in a high-binding plate (#42592, Corning), coated with the purified dtSARM1, the full-length SARM1 with the N-terminal 27-aa segment replaced by a twin-Strep and Flag-tag. The coated well was blocked with 10% BSA, incubated with the recombinant phage particles, and washed with 1% PBST for 20 times. The associated phages were released with 1 mg/mL trypsin (Thermo Fisher) and amplified by infection of *E.coli* TG1. The monoclonal phages after 3 rounds of panning were detected by Anti-M13-HRP (GE Lifesciences GE27-9421-01, 1:1000) and TMB solution (Sigma). The phagemid was sequenced in Sangon Biotech (Shanghai). The following primers were used for library construction and nanobody sequencing:

CALL001 primer: 5’-GTCCTGGCTGCTCTTCTACAAGG-3’

CALL002 primer: 5’-GGTACGTGCTGTTGAACTGTTCC-3’

VHH-Back primer: 5’-GAGTCTGGAGGAGG-3’

VHH-For primer: 5’-GGACTAGTGCGGCCGCTGGAGAC-3’

MP57 primer: 5’-TTATGCTTCCGGCTCGTATG-3’

GIII primer: 5’-CCACAGACAGCCCTCATAG-3’

### Preparation of Nbs or Nb-fusion proteins

The DNA sequence of Nb-C6, or Nb-1053 against CD38 as a negative control^26^, was amplified from the phagemid by PCR and subcloned to pET22b or pXG-mNeonGreen (from Prof. Jun Chu in SIAT/CAS, Shenzhen). The strategies for the expression and purification of the recombinant proteins were described as previously^26^. Briefly, OverExpress^TM^ *E.coli* C43(DE3) was transformed with the expression vector and grown in LB media containing kanamycin or ampicillin at 37 °C until the culture reached at OD_600_ of 1.0. Protein expression was induced by 0.5 mM IPTG at 16 °C for 20 h. Proteins were extracted by French press (ATS) at 800 psi for 3 rounds in lysis buffer (50 mM Tris pH 8.0, 500 mM NaCl) containing 1 mM PMSF and cell lysates were clarified by centrifugation at 56,000 x *g* for 45 min at 4 °C. The proteins were purified with Ni-excel resin (Cytiva) and HiTrap Q (Cytiva), sequentially, and concentrated by 10 kDa Amicon (Millipore). The following primers were used for constructing expression plasmids of Nanobody-C6:

Nb-F: 5’-ATGGCGGTGCAGCTGGTGGAGTC-3’

Nb-R: 5’-TGAGGAGACGGTGACCTGGG-3’

### Preparation of dtSARM1

The DNA sequence encoding dtSARM1, the truncated form of SARM1 without the N-terminal mitochondrial signal, fused with a twin-Strep-tag and a Flag-tag attached to the N-terminus, were subcloned into pENTR1A-GFP-N2 (#19364, Addgene), then transferred to pLenti-puro (#17452, Addgene) by Gateway^TM^ system (Thermo Fisher). The lentiviral particles were prepared by transfecting HEK293T cells with pLenti-dtSARM1, psPAX2 (#12260, Addgene), and pMD2.G (#12259, Addgene). The Expi293F cell line stably expressing dtSARM1 was established by the lentiviral infection and puromycin selection.

Then, the cells were cultured in a large volume and harvested. The dtSARM1 was released from the cytoplasm by incubating with ice-cold PBS containing 200 μM Digitonin and protease inhibitor (Roche). The cleared lysate was incubated with StrepTactin^TM^ resin (IBA) overnight at 4 °C. After washing with ice-cold Buffer W (100 mM Tris pH8.0, 150 mM NaCl, 3 mM EDTA), the proteins were eluted by 2 mM biotin in buffer W and concentrated with 100 kDa Amicon tube (Millipore). Gel filtration was performed with a Superdex 200 increase column (Cytiva) and separated the protein in aggregated and single particle forms. The concentration of dtSARM1 was determined by BCA assay (Thermo Fisher). The following primers were used for constructing expression plasmids of dtSARM1:

dtSARM1-F: 5’-CTGGCGGTGCCTGGGCCAGATG-3’

dtSARM1-R: 5’-GGTTGGACCCATGGGTGCAG-3’

### Immunoprecipitation and western blots

The lysate containing dtSARM1 was pretreated by 100 μM NMN on ice for 10 min, with non-treated dtSARM1 as a control, and incubated with StrepTactin^TM^ XT resin, in the presence of 200 ng/mL Nb-C6, or a CD38 nanobody Nb-1053^26^, overnight at 4 °C. The proteins were eluted by 2 mM biotin and analyzed by western blots with anti-His_6_ (TransGen Biotech HT501-01, 1:1000) or home-made anti-SARM1 (1:1000).

### Surface plasmon resonance (SPR)

All SPR analyses were performed with a BIAcore 8 K instrument and analyzed by BIAcore Insight evaluation software (Cytiva). To prepare the Strep-capture sensor, CM5 sensors (Cytiva) were coated with Strep-Tactin^TM^ XT protein (IBA) by amine coupling according to the manufacturer’s instructions. Briefly, all channel surfaces were activated by NHS and EDC and immobilized with Strep-Tactin XT protein diluted at 50 μg/mL in 10 mM sodium acetate, pH4.5. Excess activated groups were blocked by 1 M ethanolamine-HCl, pH 8.5. A density of Strep-Tactin XT corresponding to 3600-3800 response units (RU) was achieved.

To measure the binding affinity of Nb-C6 to SARM1, dtSARM, in the lysate of Expi293F, was captured in the sample channel to approximately 500 RU, with a blank channel as the reference channel. A series of concentrations of Nb-C6 (1.56 nM–400 nM) in the running buffer, NMN/HBS-EP (100 μM NMN, 10 mM HEPES, 150 mM NaCl, 1 mg/mL BSA, 3 mM EDTA, and 0.05% (v/v) Surfactant P20, Cytiva), were injected to both sample and reference channels for 360 s to allow association, followed by 540 s of the NMN/HBS-EP for dissociation. The equilibrium dissociation constant (*K*_D_) value was calculated from kinetics using two-state kinetics binding model on the evaluation software (Cytiva).

To correlate the Nb-C6 binding with the concentrations of NAD and NMN, the following program was conducted. After dtSARM1 was captured on the SPR sensor, HBS-EP with 2 mM NAD and 200 nM Nb-C6 was injected for 5 min to saturate the nonspecific binding and establish a baseline. HBS-EP containing 200 nM Nb-C6 and a fixed concentration of NAD (0.2, 0.4, 0.8, 1.2, 1.6 mM, each for one channel), together with an increasing gradient of NMN (0, 50, 100, 200 μM) was injected for 5 min, and washed with the same buffer without Nb-C6 for 3 min. The Nb-C6 binding was quantified by the accumulation of RU signal at the end of 30 s-washing step and normalized by those in the condition of 200 μM NMN (representing 100% binding). The Nb-C6 binding percentage and molar ratio of NMN and NAD were plotted.

### Immunostaining and imaging SARM1 with Nb-C6

HEK293 cells carrying the inducible expression cassette for SARM1 were seeded on coverslips precoated with 0.05 mg/mL poly-l-lysine. After 12-h induction with 1 μg/mL doxycycline and 6-h activation with 100 μM CZ-48, the cellular SARM1 was immunostained by Nb-C6 as the following procedure. The cells were fixed in 1% PFA for 15 min and permeabilized with 0.1% Triton X-100 in PBS for 5 min. After blocking with 10 mg/ml BSA for 1 h, the cells were incubated with C6-mNeonGreen, anti-Tom20 (Santa Cruze sc-17764, 1:1000), and anti-SARM1 (home-made, 1:1000) at room temperature for 1 h. The fluorescence was developed by incubating with the Alexa Fluor^TM^-conjugated secondary antibodies (Thermo Fisher A10042/A31571, 1:1000), and imaged under a confocal microscope (Nikon A1R) with a 60× object, and analyzed by NIS-Elements AR analysis (Nikon). ImageJ and Imaris Bitplane software were used for the colocalization analysis of the confocal signals.

### Reverse cycling assay to measure the ADP-ribosyl cyclase activity

The endogenous NAD in dtSARM1-containing HEK293 lysate was removed by ultrafiltration with 10 kDa Amicon tubes (Millipore) by a dilution factor of more than 1,000,000. The cleaned lysate, containing dtSARM1 was incubated with 10nM Nb-C6, or Nb-1053 as negative control, with or without 100 μM NMN on ice for 1 h separately. The reverse cycling reaction was conducted as described^36^. Briefly, 100 μL of reaction systems were prepared in PBS buffer, containing 80 μg of the cleaned lysate, 10 μM cADPR and 100 μM nicotinamide. After incubation for the indicated period at room temperature, the reaction was stopped by adding the same volume of 200 mM HCl and subsequently neutralized by 2 M Tris pH8.0. The amount of the produced NAD was measured by the cycling assay^37^ and the enzymatic activity was characterized by the initial reaction velocity, pmol NAD/h and normalized by the reaction velocity catalyzed by the NMN-activated SARM1 sample.

### Quantification of cellular cADPR in cells expressing Nb-C6

HEK293 cells carrying the inducible SARM1 expression cassette were transiently transfected with the plasmids encoding the YFP-fusion nanobody, C6-YFP or 1053-YFP, by PEI. Forty-eight hours post-transfection, the cells were lysed with 0.6 M perchloric acid. After centrifugation, the pellets were re-dissolved in 1 M NaOH and quantified by Bradford assay (Quick Start^TM^ Bradford Kit, BIO-RAD), and the supernatants containing cADPR were neutralized with Chloroform/Tri-n-octylamine mixture (volume ratio 3:1) and treated with NADase (home-made). The concentration of cADPR was analyzed by the cycling assay as described previously^37^. The results were presented as pmol cADPR/mg proteins. The protein expression level was quantified by western blot with Anti-GFP (TranGen HT801-01, 1:1000).

### Cryo-EM sample preparation and data acquisition

Three mg/mL of pure dtSARM1 was incubated with 100 µM NMN in a binding solution (100 mM Tris (pH 8.0), 150 mM NaCl, and 1 mM EDTA) on ice for 10 min. Nb-C6 was then added at 1:1 molar ratio with dtSARM1. After 30 min incubation on ice, the sample was applied to glow-discharged M026-Au300-R20/20 Copper grids (CryoMatrix). The samples were subsequently blotted for 3 s at 8 °C with 100% humidity and plunge-frozen in a VitroBot Mark IV (Thermo Fisher).

Data were collected using a 300 kV, K2 Summit (Gatan)-equipped Thermo Fisher Titan Krios electron microscope. The nominal magnification was set at 130,000X, which corresponds to a calibrated pixel size of 1.076 Å, and the gatan imaging filter was set with a slit width of 40 eV. Data were automatically acquired using SerialEM with defocus ranging from −0.8 to −2.5 μm. A total of 50 electrons/Å^2^ were distributed over 39 frames in each movie.

### Cryo-EM image processing

4,605 movies obtained in super-resolution mode were subjected to motion correction using the MotionCor2 integrated into relion-3.1.0 with dose-weighting. 3,609 micrographs were manually chosen based on CTF calibration with CTFFIND4.1 (Relion). 3,089,111 particles in total were automatically selected using the Laplacian-of-Gaussian approach. 2,384,641 particles were chosen after several rounds of 2D and 3D classification in Relion and exported into cisTEM-1.0.0 for 3D classification and refinement. A final particle stack of 208,299 particles was chosen. The final reconstruction map displayed an overall resolution of 2.7 Å, but the density near the ARM and TIR region was relatively poor (Supplementary Fig. 4a).

The particle stack was exported into relion-3.1.0 and extended with D8 symmetry using the relion_particle_symmetry_expand module. Expanded particles were subtracted using a mask that excluded all but one TIR-ARM component. One round of 3D Classification without mask was performed to remove the particles with poor TIR-ARM densities, after which, the remaining 756,091 particles were subjected to focused 3D classification with a limited mask around the ARM-TIR region in order to obtain higher resolution around the TIR domain and ARM domain’s outer region. Three major classes were separated in this round, two of them displayed clear densities for outer part of ARM domain (corresponding to ARM1-4 helices) and the third class was dim and inaccurate around the ARM-TIR domain (Supplementary Fig. 4e). The 298,862 particles in Class 1 and 133,097 particles in Class 2 were chosen for 3D refinement with C1 symmetry, which yield two density maps of resolution 3.3 Å and 3.4 Å for Class 1 and Class2, respectively (Supplementary Figs. 3c and 4c, d).

The multi-body refinement method^28^ was employed to increase the local resolution of TIR and ARM domains, which are flexible in structure. Both Class 1 and Class 2 densities were divided into two rotating bodies, one small body containing one TIR-ARM-SAM/Nb-C6 unit and one large body containing the remaining components. For the Class 1 dataset, the refinements converged after 13 iterations and reconstructed maps for the big and small bodies of 3.3 Å and 3.7 Å respectively. For the Class 2 dataset, the refinements converged after 13 iterations and reconstructed maps for the big and small bodies of 3.4 Å and 3.8 Å, respectively. The final maps of the small bodies, which were evaluated using Resmap^38^, exhibits similar local resolution of ~3.0 Å around the SAM domain, 3.6–4.2 Å for the TIR-ARM domains of Class 1, 3.6-4.5 Å for the TIR-ARM domains of Class 2 (Supplementary Fig. 4c and 5a, b).

### Model building and structure refinement

The SARM1^NMN^ models were constructed based on a starting model of SARM1 binding to the inhibitor dHNN (PDB 7DJT)^23^. Using the Dock in Map module of Phenix 1.16, the SAM1-SAM2 region (403–549), the ARM domain (60–402), and the TIR domain (561–701) were each separately docked into the refined map and then manually modified in COOT 0.9. The Nb-C6 structure starting model was created and adjusted based on another Nb with a similar overall structure (PDB 5F1K)^26^ using COOT 0.9. The modified model was then manually adjusted in COOT 0.9 after each round of refinement using the Real-space refinement module of Phenix 1.16. The statistics for Cryo-EM data collection and structure refinement are summarized in Supplementary Table 2.

### Analysis of the activity of SARM1 mutants

The mutants of SARM1, including W103A, R110A, K193M, D317A, D317R, E189Q, L257C, S319F, S319Y, Q320A, Q320Y, and F476C were produced by introducing the mutations into the full-length DNA sequence of SARM1 by overlap extension PCR. The resulting mutant genes were subcloned to pENTR1A-GFP-N2 and transferred into the lentivector, pInducer20 (#44012, Addgene) by Gateway system. HEK293 cell line stably expressing SARM1 mutants were established by lentiviral infection and puromycin selection. After induction by 1 μg/mL doxycycline for 24 h, the mutants were extracted with PBS containing 4 mM digitonin and protease inhibitors (Roche).

The activities of mutants were analyzed with PC6 assay with or without pre-activation by 100 µM NMN or acid, as described previously^39^. For the acid treatment, the mutants were incubated with 0.2 M CH_3_COONa (pH 4.3) for 30 s and neutralized with 2 M Tris-HCl (pH8.0). The kinetics of fluorescence (Ex390/Em520) production catalyzed by SARM1 was measured after adding the reaction reagents containing 50 μM PC6 and 100 μM NAD in 50 mM Tris-HCl (pH 7.5) on a Synergy H1 Hybrid Reader (BioTek). The initial rate (RFU/min) of the reactions were calculated to quantify the activities of SARM1. The same amounts of lysates were used for western blots.

### Hydrogen-deuterium exchange mass spectrometry (HDX-MS)

HDX was performed by pre-incubating 2 µM dtSARM1 with 2 mM NAD or 200 µM NMN at 28 °C for 10 min. After equilibration, H/D exchange was carried out by 10-fold dilution into a D_2_O buffer (25 mM HEPES, pHread 8.0, 150 mM NaCl) to a final volume of 100 uL. D_2_O buffer was supplemented with 2 mM NAD or 200 µM NMN. After 10 s, 30 s, 1 min, 5 min, and 30 min of incubation at 28 °C, the reaction was quenched by addition of ice-cold quench solution (1:1, v/v) containing 2 M guanidinium hydrochloride and 0.2 M citric acid, resulting in a final pH of 2.2. This preparation was digested on an immobilized pepsin column inside a manual HDX-UPLC system (UltiMate 3000, Thermo Fisher) with the temperature maintained at 0 °C (ice-cold). Eluted peptides were desalted using chilled trap column (1 mm × 15 mm, Acclaim PepMap300 C18, 5μm, Thermo Fisher Scientific) for 5 min at a flowrate of 200 μL/min and 0.1% formic acid as mobile phase. Subsequent peptide separation was performed on the chilled ACQUITY BEH C18 (2.1 x 50 mm) analytical column using a first gradient ranging from 9 to 45% of buffer B (80% acetonitrile and 0.1% formic acid) for 10 min followed by a second gradient ranging from 45 to 99% of buffer B for 1 min, at an overall flow rate of 50 μL/min. Peptides were ionized via electrospray ionization and analyzed by Orbitrap Eclipse (Thermo Fisher) mass spectrometer. Mass spectra were collected in triplicate for each exchange period. Non-deuterated samples peptide identification was performed via tandem MS/MS experiments and analyzed by Proteome Discoverer 2.5 (Thermo Fisher). A full MS scan was collected in the Orbitrap (m/z: 300–1500, resolution: 60,000, AGC 4e5, max injection time: 50 ms) followed by a series of MS/MS scans measured in the Orbitrap (HCD normalized collision energy: 30%, resolution: 15,000, AGC 5e4, isolation width 1.6 m/z, max injection time: AUTO). Unspecific enzyme cleavage was used for all searches. Search results were filtered by requiring precursor tolerance (±10 ppm) and fragment tolerance (±0.02 Da). Searches were performed against a database containing the sequence of SARM1. For the deuterated mass spectra, a full MS scan was collected in the Orbitrap (resolution: 60,000, AGC 4e5, max injection time: 50 ms). Mass analysis of the peptide centroids was carried by HDExaminer v3.3 (Sierra Analytics, Modesto, CA), followed by manual verification for each peptide. No corrections for back exchange that occurs during digestion and LC separation were applied. When computing deuteration percentages for uptake plots and the heat map, the 100% level for peptides was defined to be the theoretical maximum deuteration level. For peptides exhibiting a bimodal distribution all intensity values belonging to one incubation time in D_2_O were analyzed by fitting two Gaussian peaks with different means and areas but with similar width into the spectra, as described earlier^40^. Next, the fitted peak area parameter was used to calculate the relative amount of open state by taking the ratio of high and the sum of high and low mass subpopulations. The reported pHread values are direct pH meter readings of the D_2_O buffer solutions calibrated with standard buffer solutions made with H_2_O and are uncorrected for the isotope effect at the glass electrode^41^.

### Crosslinking mass spectrometry (XL-MS)

Crosslinking sample preparation was started with pre-incubating dtSARM1 (in 20 mM HEPES pH8.4) with 2 mM NAD or 200 µM NMN on ice for 10 min. Ligand binding SARM1 was incubated with DSBU crosslinker (Thermo Fisher) to a final concentration of 200 µM for 30 min at 25 °C, and quenched with 10 mM ammonium bicarbonate. The proteins were denatured with 10 mM DTT and 8 M urea for 60 min, then alkylated by 50 mM chloroacetamide (CAA) for 30 min in dark. Then the protein was digested by trypsin (trypsin: protein = 1:20, w/w) overnight at 37 °C. Tryptic peptides were desalted using Pierce peptide desalting spin columns and loaded (700 ng in 0.1% formic acid) onto a nano-trap column (75μm i.d. × 2 cm precolumn, packed with Acclaim PepMap100 C18, 3 μm, 100 Å; Thermo Fisher) using EASY-nLC 1200 system (Thermo Fisher). Subsequent separation was performed on the analytical column (50 μm × 15 cm, Acclaim PepMap RSLC C18, 2 μm, 100 Å; Thermo Fisher Scientific) using a first gradient ranging from 2 to 8 % of buffer B (80% acetonitrile and 0.1% formic acid) for 5 min followed by a second gradient ranging from 8 to 43% of buffer B for 80 min, and third gradient ranging from 43 to 50% of buffer B for 5 min at an overall flow rate of 300 nL/min. Peptides were ionized via nano-electrospray ionization and analyzed by Orbitrap Eclipse mass spectrometer (Thermo Fisher) using sceHCD-MS2 fragmentation approach^42–44^. Survey scans were recorded in the Orbitrap at 60,000 resolution (AGC 4e5, max injection time 50 ms) and a scan range from 350 to 1600 m/z. MS/MS scans were recorded in the Orbitrap at 30,000 resolutions (AGC 1e5, max injection time 120 ms, isolation width 1.6 m/z). Unknown, singly and doubly charged ions were excluded from fragmentation. Selected precursors were fragmented by applying a stepped-HCD energy of 30 ± 3% NCE. Proteome Discoverer 2.5 (Thermo Fisher) with XlinkX software 2.5 was used to analyze and identify the crosslinking peptides and the parameters were as follows: maximum of three missed cleavage sites for trypsin per peptide; cysteine carbamidomethylation as fixed modification and methionine oxidation as dynamic modification. Searches were performed against an ad-hock database containing the sequence of SARM1 and common contaminant proteins (CRAPome/Strep tag AP^45^). Search results were filtered by requiring precursor tolerance (±10 ppm) and fragment tolerance (±20 ppm). FDR threshold was set to 1% at Crosslink and CSM level. The mass spectrometry proteomics data have been deposited to the ProteomeXchange Consortium via the PRIDE^46^ partner repository with the dataset identifier PXD033528.

### Data analysis

All experiments contained at least three biological replicates. Data shown in each figure are all means ± SD. The unpaired Student’s *t*-test was used to determine the statistical significance of differences between means (*P < 0.05, **P < 0.01, ***P < 0.001, ****P < 0.0001). GraphPad Prism 8.0.1 was used for data analysis.

### Reporting summary

Further information on research design is available in the Nature Portfolio Reporting Summary linked to this article.

## Supplementary information

Supplementary Information

Description of Additional Supplementary Files

Supplementary Data 1

Supplementary Data 2

Supplementary Data 3

Supplementary Data 4

Reporting Summary

## Data Availability

Atomic coordinates for SARM1/NMN/Nanobody-C6 have been deposited in the wwPDB under accession codes 8GQ5 (Overall Structure), 8GNI (TIR-ARM Conformation 1), and 8GNJ (TIR-ARM Conformation 2), respectively. Cryo-EM densities have been deposited in the Electron Microscopy Data Bank (EMDB) under accession codes EMD-34198 (Overall Structure), EMD-34165 (TIR-ARM Conformation 1), and EMD-34166 (TIR-ARM Conformation 2), respectively. The mass spectrometry proteomics data have been deposited to the ProteomeXchange Consortium via the PRIDE^46^ partner repository with the dataset identifier PXD033528. Source data are provided with this paper. Protein structure models shown in Supplementary Fig. 8 were generated with SWISS-MODEL (https://swissmodel.expasy.org/) using PDB 7ANW (hSARM1 NAD + complex) as template; the model coordinates are provided as Supplementary Data 3 and 4. Other structures previously published are also available from the PDB under accession codes 7CM6 (NAD + -bound Sarm1 in the self-inhibited state), 7DJT (Human SARM1 inhibitory state in complex with inhibitor dHNN), 5F1K (Human CD38 in complex with nanobody MU1053), 7NAG (TIR domain in complex with 1AD), 7NAH (TIR domain in complex with 2AD), 7NAI (TIR domain in complex with 3AD), 7NAJ (TIR domain in complex with ara-2’F-ADPR), 7NAK (Human SARM1 in complex with NMN and 1AD) and 7NAL (Human SARM1 in complex with NMN and 1AD). Source data are provided with this paper.

## Source data

Source Data
