# Supplementary material for: A conformation-specific nanobody targeting the nicotinamide mononucleotide-activated state of SARM1
Source: Nat Commun. 2022 Dec 22;13:7898. doi: 10.1038/s41467-022-35581-y (PMC9780360; doi:10.1038/s41467-022-35581-y)
Supplement: Supplementary file 1 — Supplementary Information [file 41467_2022_35581_MOESM1_ESM.pdf]

A Conformation-specific Nanobody Targeting the NMN-activated State of SARM1

Yun Nan **Hou**[1#], Yang **Cai**[2#], Wan Hua **Li**[1,3#], Wei Ming **He**[1], Zhi Ying **Zhao**[1], Wen Jie **Zhu**[1], Qiang **Wang**[1], Xinyi **Mai**[4], Jun **Liu**[1], Hon Cheung **Lee**[1], Stjepanovic **Goran**[4*], Hongmin **Zhang**[2*], Yong Juan **Zhao**[1,3*]

[1] State Key Laboratory of Chemical Oncogenomics, Key Laboratory of Chemical Genomics, Peking University Shenzhen Graduate School, Shenzhen, China, 518055

[2] Department of Biology, School of Life Sciences, Southern University of Science and Technology, Shenzhen, China, 518055

[3] Ciechanover Institute of Precision and Regenerative Medicine, School of Medicine, The Chinese University of Hong Kong, Shenzhen, China, 518172

[4] Kobilka Institute of Innovative Drug Discovery, School of Medicine, The Chinese University of Hong Kong, Shenzhen, China, 518172

[#] Equal contribution

* Corresponding authors: goranstjepanovic@cuhk.edu.cn; zhanghm@sustech.edu.cn; zhaoyongjuan@cuhk.edu.cn

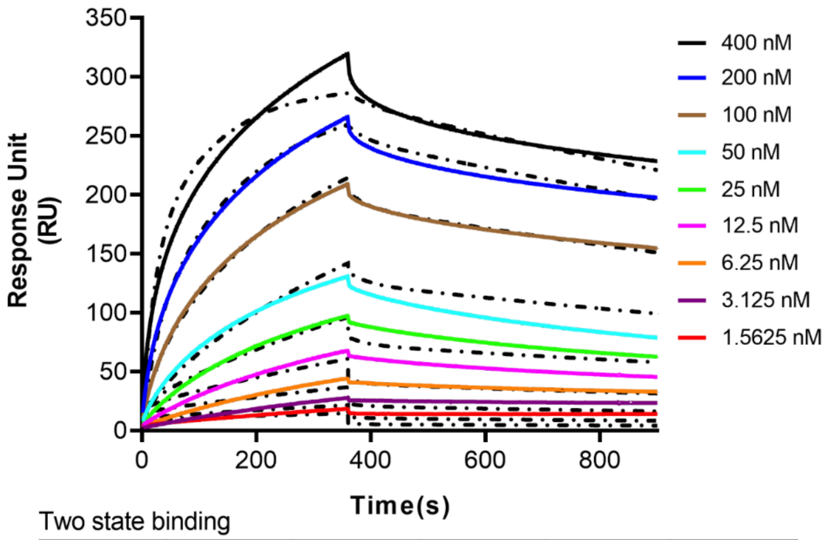

Two state binding

	k_a1 (1/Ms)	k_d1 (1/s)	k_a2 (1/s)	k_d2 (1/s)	K_D (M)
Average	4.84E+04	2.09E-02	1.02E-02	6.01E-04	2.47E-08
SD	1.46E+03	8.96E-04	4.33E-04	5.55E-05	3.04E-09

Supplementary Figure 1. SRP analysis for the affinity between Nb-C6 and SARM1. The recombinant dtSARM1 was immobilized on SPR sensor through the interaction between twin-strep tag and StrepTactin™ XT. A series of concentrations of Nb-C6 (1.56 nM-400 nM) in the running buffer HBS-EP containing 100 μM NMN were injected to both sample and reference channels for 360 s to allow association, followed by 540s of NMN/HBS-EP for dissociation. The data were analyzed with the BIAcore Insight evaluation software (Cytiva), giving the association rate constants (k_a1 and k_a2), dissociation rate constants (k_d1 and k_d2) and equilibrium constant K_D as listed in the table. The black dash curves are the fitting curves using two-state model. The data shown is the representative of 3 independent experiments.

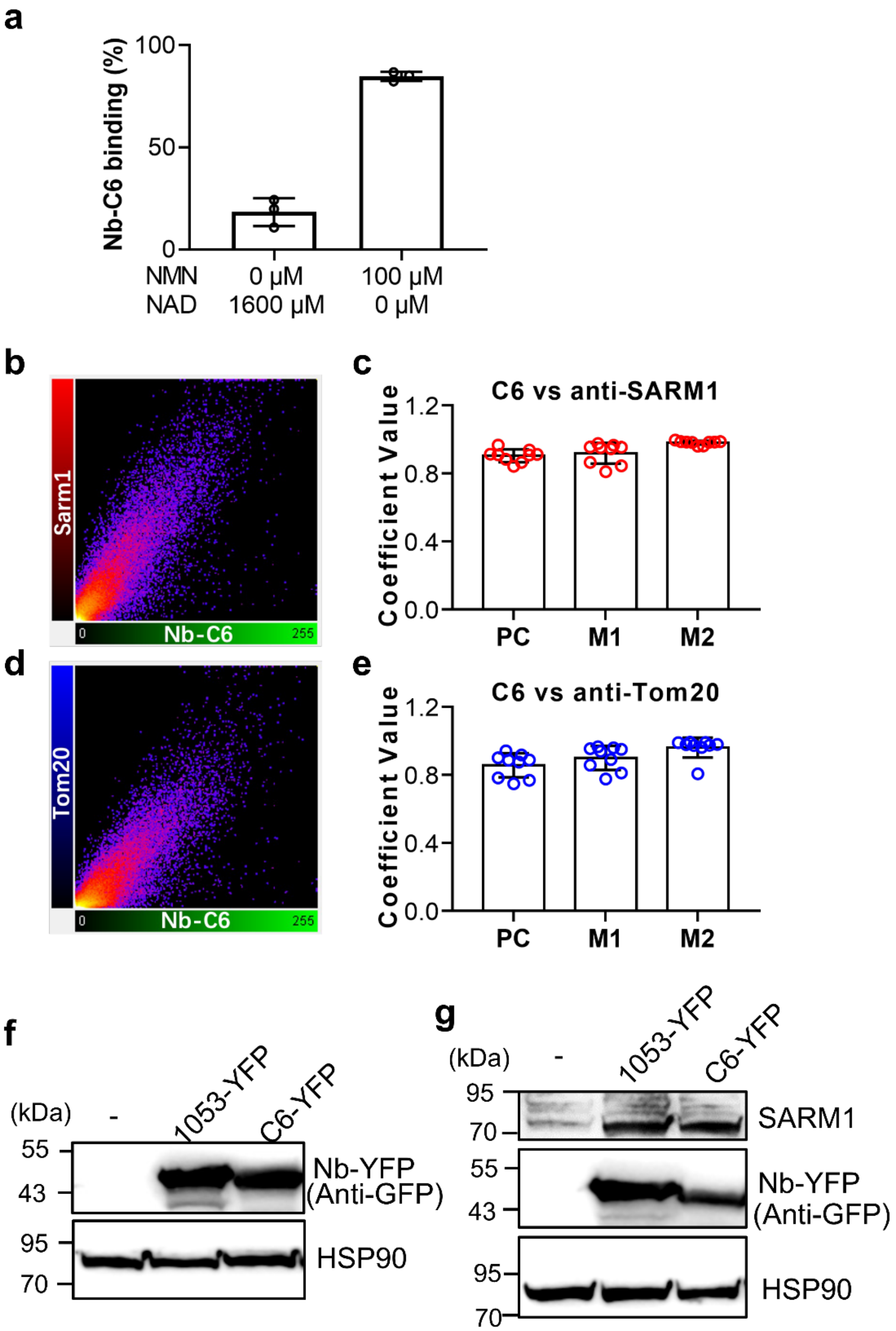

Supplementary Figure 2. Supplementary data for Fig. 2. a From the original data in **Fig. 2a**. The Nb-C6 binding percentage and the concentrations of NMN and NAD in two conditions were plotted in a bar graph. The Nb-C6 binding was normalized to the binding at 200 uM NMN, set as 100%. (n=4 biological independent experiments, mean ± SD) **b-e** Co-localization analysis of Nb-C6 and anti-SARM1 or anti-Tom20. The pictures are from Fig. 2c (CZ-48 panel). (b and d) Colocalization analysis with Imaris software. Scatter-plot pixels correspond to the images shown in Fig. 2b. Complete colocalization results in a pixel distribution along a straight line whose slope will depend on the fluorescence ratio between the two channels. (c and e) PC, M1,

and M2 were analyzed with JACoP. M1 is defined as the fraction of Nb-C6 overlapping anti-SARM1 (red) or anti-Tom20 (blue) signals; M2 is defined conversely. (n=55 cells in 10 images examined over 3 biologically independent samples, mean ± SD)
f-g Protein expression for Fig. 6d and 6e tested by western blots. (n=3 biological independent experiments)

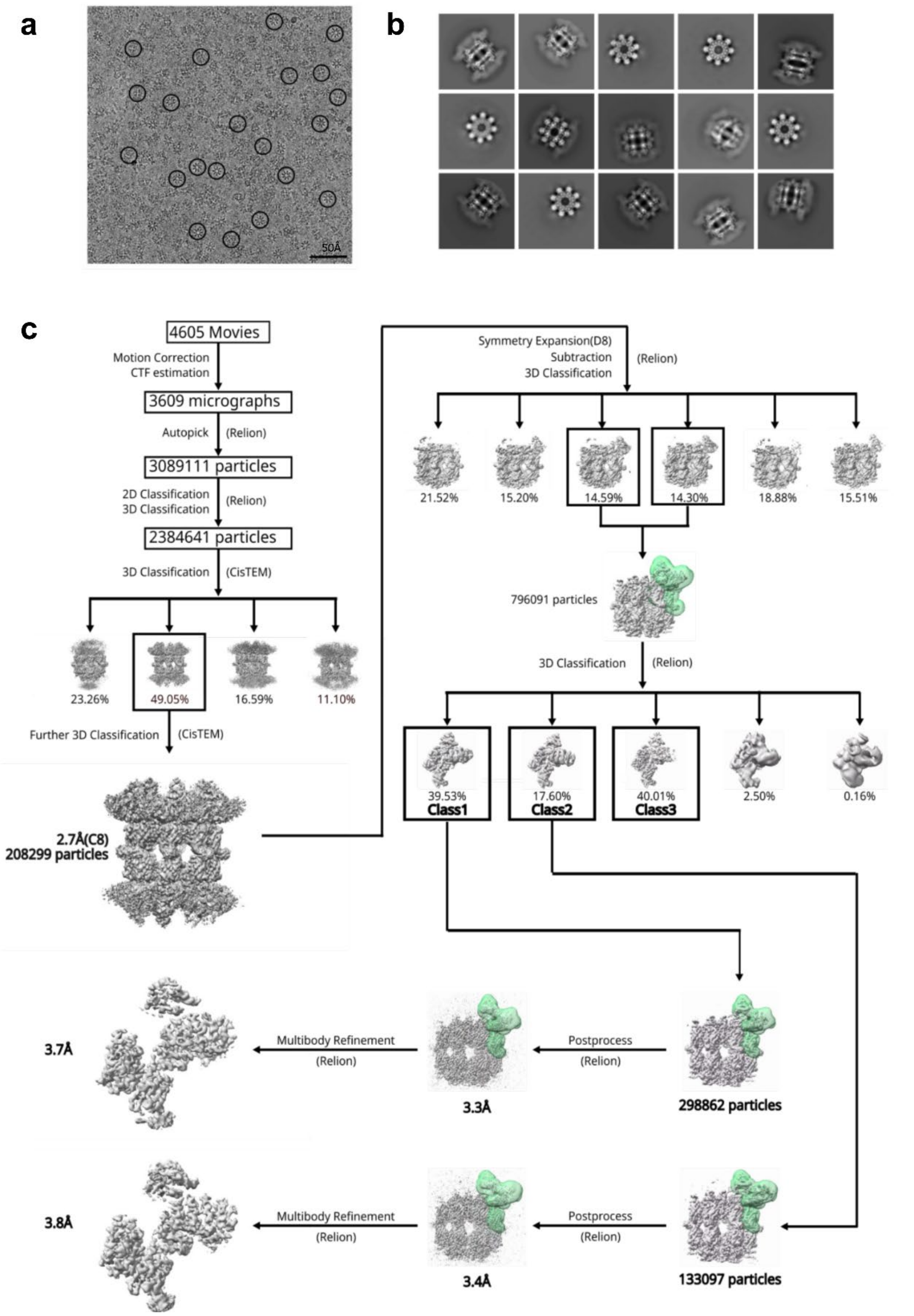

Supplementary Figure 3. Imaging processing procedure of SARM1^{NMN}/Nb-C6 complex. **a** Raw cryo-EM image displaying particles of SARM1^{NMN}/Nb-C6 complex in different directions. Several representative particles of different projective directions were circled. The data shown is the representative of 4605 independent movies. **b** Selected 2D class averages showing both top and side views of the SARM1^{NMN}/Nb-C6 complex. **c** Image-processing workflow for 3D refinement and

reconstruction of SARM1^{NMN}/Nb-C6 complex. 3D Refinement and reconstruction with C8 symmetry group yielded a density map of 2.7Å. The dataset was then expanded with D8 symmetry group and all but one TIR-ARM unit were subtracted. Three major classes were selected based on focused 3D classification with a green mask around ARM-TIR domains, which are identified as Class1,2 and 3. Better local resolution of the ARM and TIR domain was obtained by multibody-refinement with masked areas which contains only one unit of ARM-SAM/TIR/Nb-C6 for both Class 1 and Class 2, generated a 3.7Å map for Class1 and a 3.8Å map for Class 2.

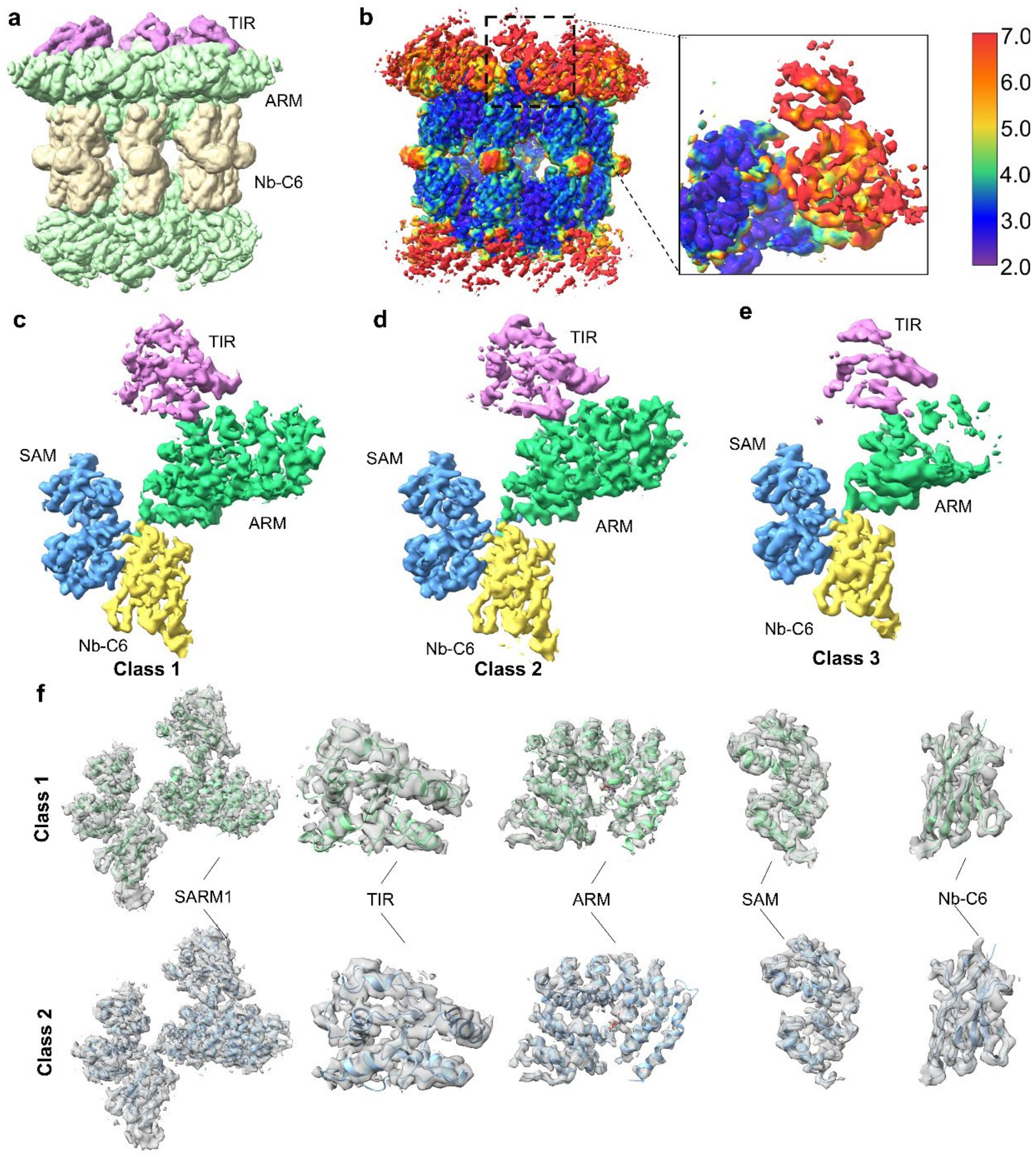

Supplementary Figure 4. Depiction of density maps of SARM1^{NMN}/Nb-C6 complex. **a** The Two-layer octameric structure of SARM1, bridged by Nb-C6. **b** Local resolution of the double-layer model, the density around TIR and ARM domains were of relatively low resolutions as a result of the flexibility of ARM-TIR domains. **c-e** Three major classes separated by focused 3D refinement with mask limited to TIR-ARM domain. The model of Class 1 and Class 2 displayed clear densities around TIR and ARM domain, especially ARM1-4 helixes. The density around TIR-ARM domains in Class 3 was vague and fragmentary. **f** Model fitting of Nb-C6 and each domain of SARM1 to density of Class 1 and Class 2, respectively.

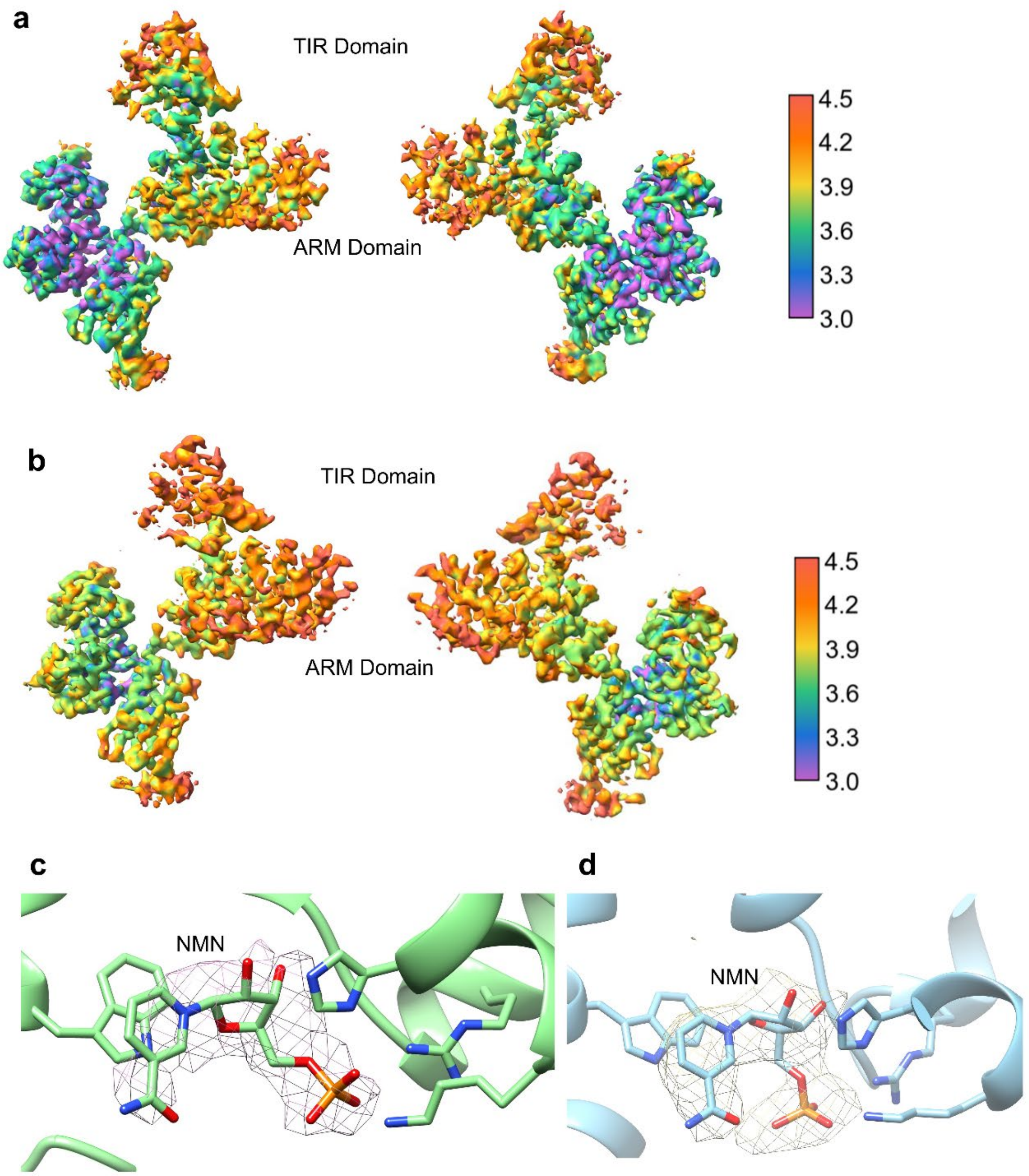

Supplementary Figure 5. Local Resolution of Density maps of Class 1 and Class 2. a-b Local Resolution of Class 1 (a) or Class 2 (b) analyzed by ResMap. **c-d** NMN locally fitted in corresponding densities of Class 1 (c) or Class 2 (d), within the binding pocket of ARM domain.

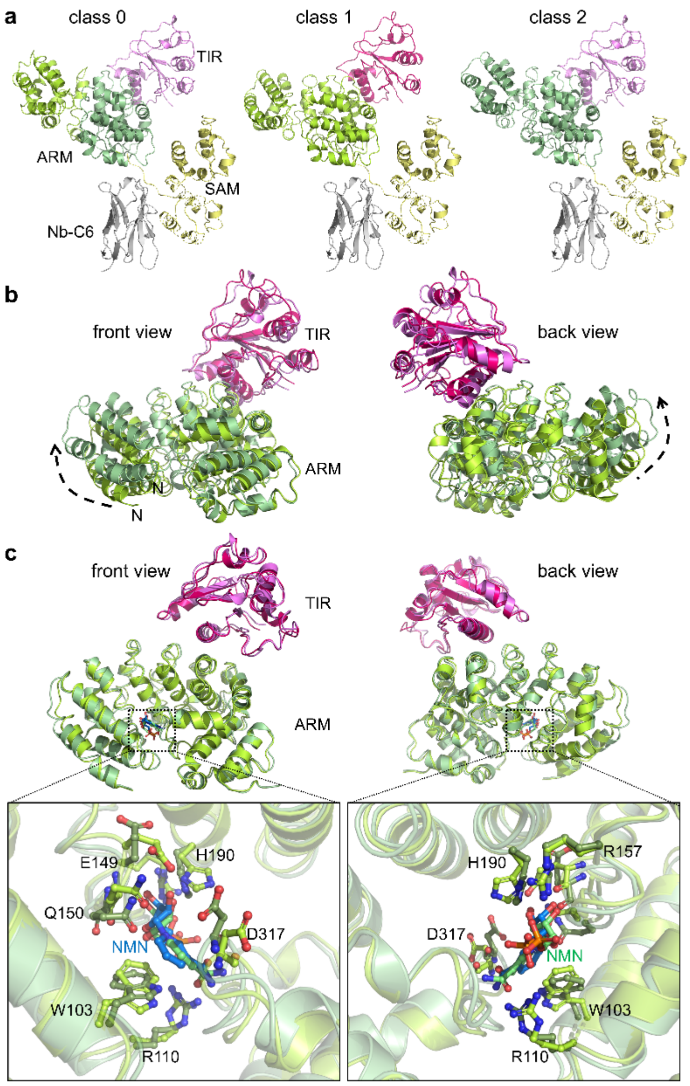

Supplementary Figure 6. Two conformations of ARM-TIR domains observed in the SARM1^{NMN}/C6 structure. a Cartoon presentation of the conformations observed for one SARM/C6/NMN protomer. Class 0 is the model fit into the original mixed-density while class 1 and class 2 are two conformations separated from the mixed-density. The ARM, SAM, TIR and nanobody C6 are labeled. **b** Superposition of the two conformations via the SAM and nanobody. For clarity, the SAM and nanobody C6 are not shown. The ARM domains and TIR domains of class 1 and class2 are shown in limon green, pale green, hot pink and violet respectively. The ARM and TIR domains of class 1 rotate clock wisely about 5° as indicated by a black arrow. **c** The Superposition of the ARM domains from class 1 and class 2. The ARM and TIR domains are colored as in panel b. The NMN binding sites in both conformations are shown in zoomed-in view. NMN and residues interacting with NMN are shown in ball-and-stick models.

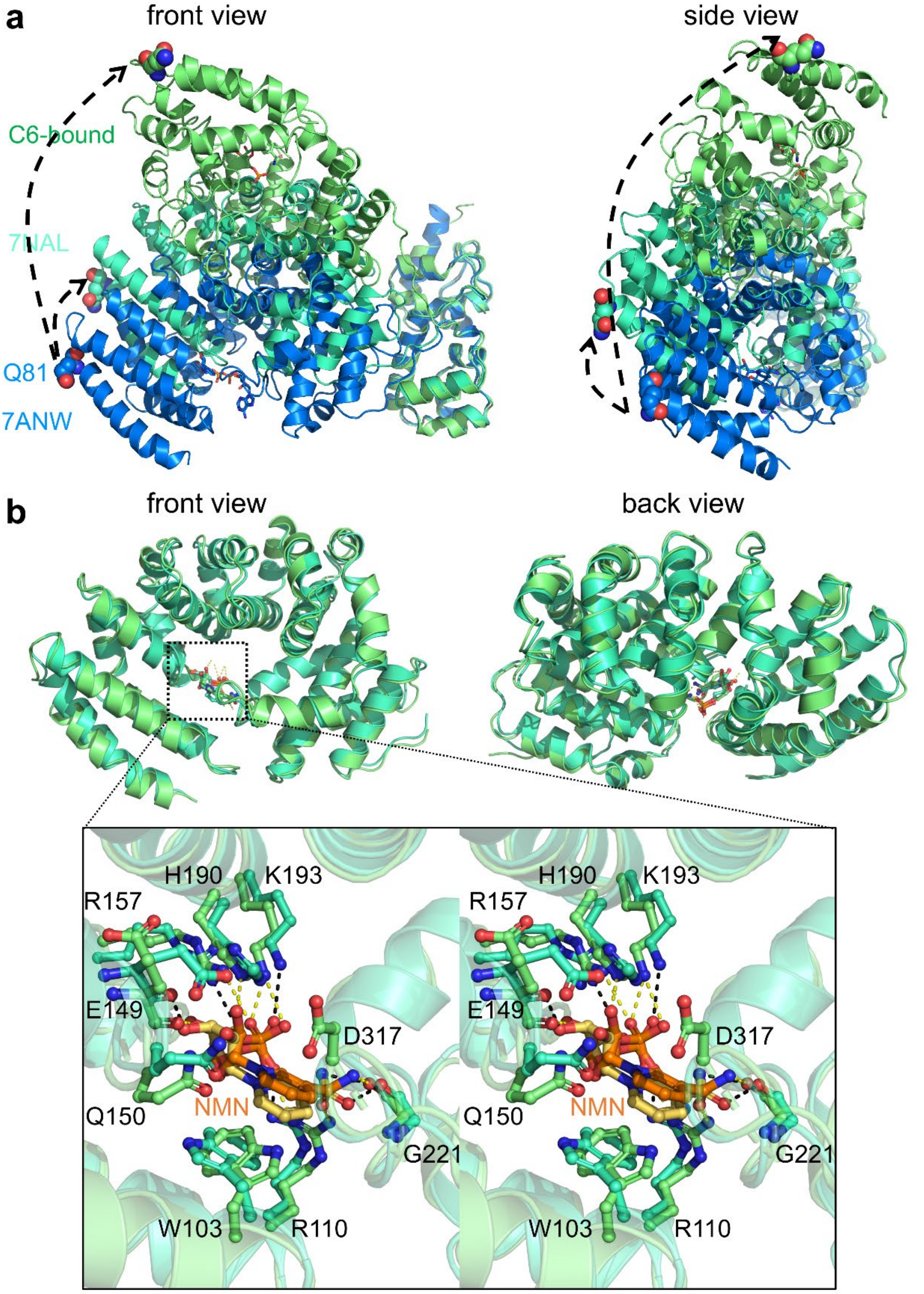

Supplementary Figure 7. Comparison between the ARM domains of SARM1^{NMN} (PDB 7NAL) and SARM1^{NMN}/Nb-C6 (this work, class 2). a Superposition via the SAM domains of SARM1^{NAD} (7ANW), SARM1^{NMN} (7NAL) and SARM1^{NMN}/Nb-C6 (this work) revealed the rotation of the ARM domains. Residue Q81 was shown in sphere mode as reference of the conformational change. **b** Superposition of SARM1^{NMN} (7NAL and this work) indicated almost identical conformation between these two structures. A zoom-in stereo view showed the NMN-binding sites. 7NAL was colored

in cyan-green while our structure in light green. NMN molecules in 7NAL and our structure were colored in pale-yellow and orange, respectively. Hydrogen bonds were indicated as dash lines in black (7NAL) and yellow (this work), respectively.

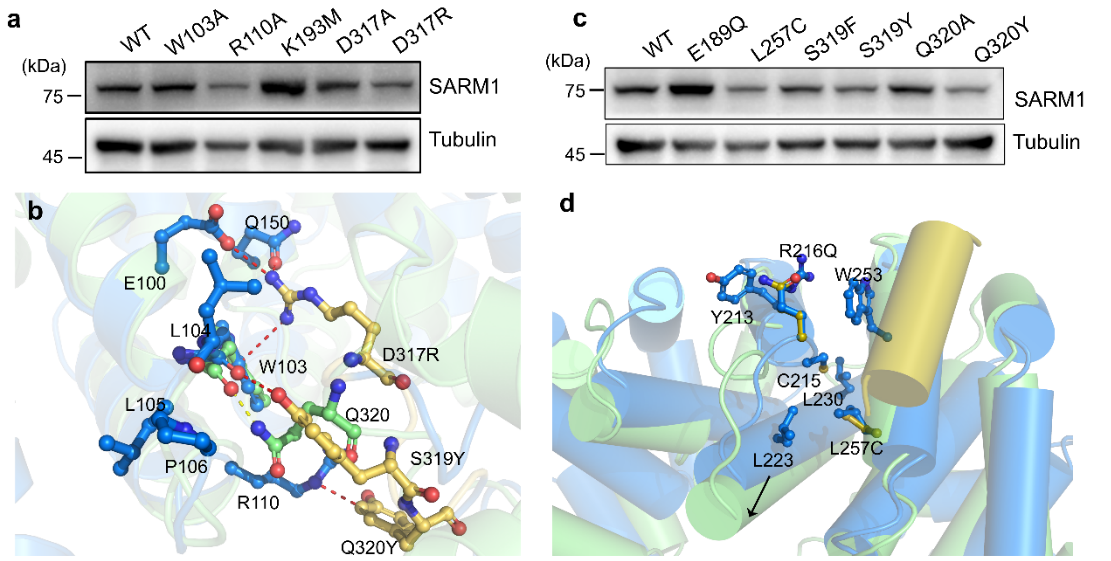

Supplementary Figure 8. Residues contribute to the NMN-induced conformational change of the ARM domain. a,c Protein expression for Fig. 4c and 4f tested by western blots. (n=3 biological independent experiments) **b** Modeling of D317 loop. NMN- and NAD-bound ARM domains (PDB 7ANW) were superimposed via helix 95-105. The color codes are the same as panels Fig. 4a-b. D317R, S319Y and Q320Y in ARM^{NAD} were modelled to inspect the interaction with surrounding residues (coordinates shown in Supplementary Data 3). D317R forms salt bridge with the side chains of residues E100 and Q150 and also positive charge-pi interaction with residue W103, which would stabilize the inactive conformation of the ARM domain. D317E mutant should have similar interaction with H150. S319Y would form hydrophobic interaction with residues L104, L105 and P106 and also H-bond with main chain carboxylate of W103. S319F mutant should form similar interactions to that of S319Y. Q320Y mutant might form charge-pi interaction with residue R110. The D317R, S319Y/F and Q320Y mutants would stabilize the inactive conformation of ARM domain, preventing the inward movement of D317 loop and consistent with the enzymatic results. In ARM^{NMN}, the side chain carboxlamine of Q320 forms H-bond with the main chain carboxylate of residue W104. Q320A mutant should diminish the H-bond and weaken the NMN-activation, consistent with the enzymatic result. **d,** Modelling of mutations at R216 and L257. The ARM-TIR domains of NMN-bound (shown in green) and NAD-bound SARM1 (shown in blue) were superimposed via the helix from AA 575-585 (shown in yellow). The shift of helix from AA 221-236 was indicated by a black arrow. R216Q and L257C mutants were modelled and shown in gold (coordinates shown in Supplementary Data 4). In SARM1^{NAD}, R216 might form pi-Arg-pi interaction with Y213 and W253 and even salt-bridge with residue E689 in the TIR domain. R216Q mutation diminished all these interactions. L257C mutation weakened the hydrophobic interaction with residues L223 and L230 in the helix (221-236), and might even form disulfide bond with C215, facilitating the inward shift of the helix (221-236) induced by NMN. The enzymatic measurements of R216Q and L257C mutants are consistent with the modeling results. The modelling in (b) and (d) was done by Swiss-model online server with 7ANW as a template.

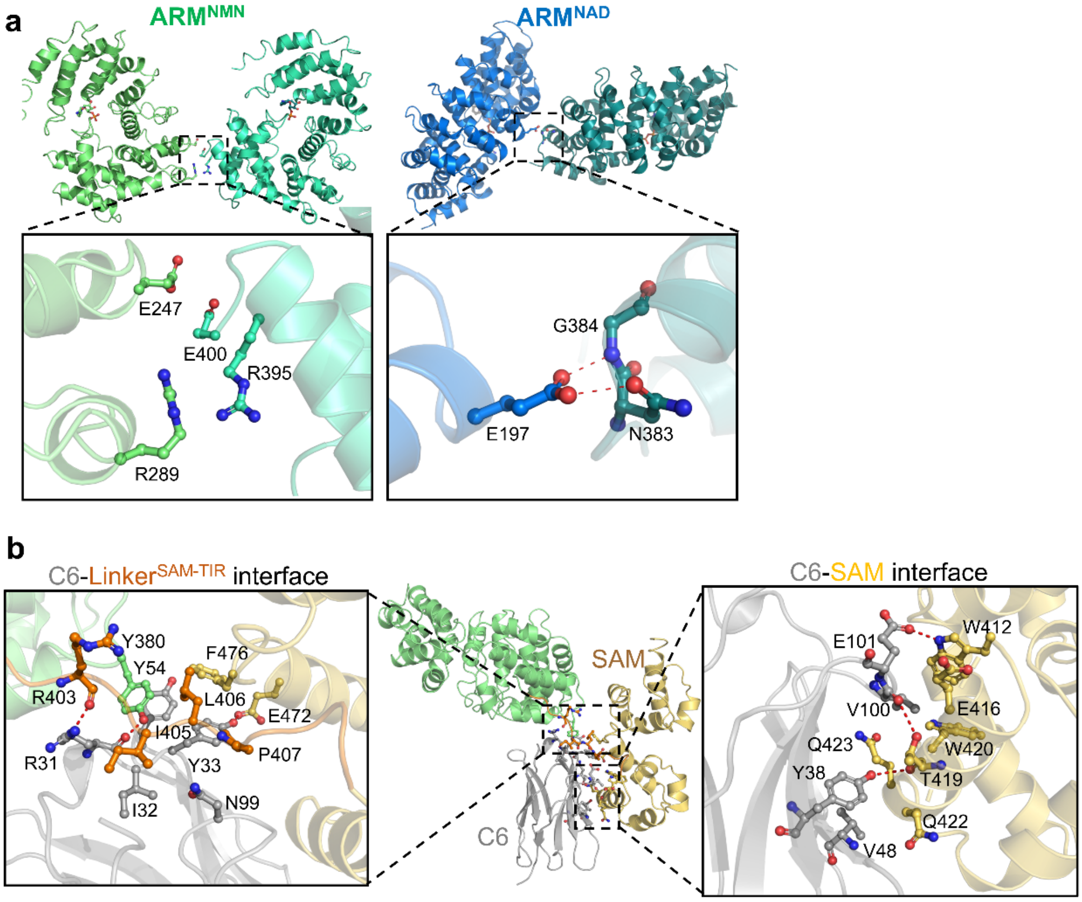

Supplementary Figure 9. Interfaces between Nb-C6 and SARM1 or ARM and ARM domains. a Comparison of the ARM-ARM interface in SARM1^{NMN} (left panel) and that in SARM1^{NAD} (PDB 7CM6) (right panel). The adjacent ARM domains in both SARM1 molecules were shown as cartoon models and colored differently. The residues at the interface were shown as ball-and-stick models. **b** Interfaces between Nb-C6 and SARM1. Nb-C6 and SARM1 are shown as cartoon models and colored differently for each domain (grey for Nb-C6, green for the ARM domain, yellow for the SAM domain and gold for the ARM-SAM linker). Key interaction residues were shown as ball-and-stick models and labeled. The H-bonds and salt-bridge were shown as red dash lines.

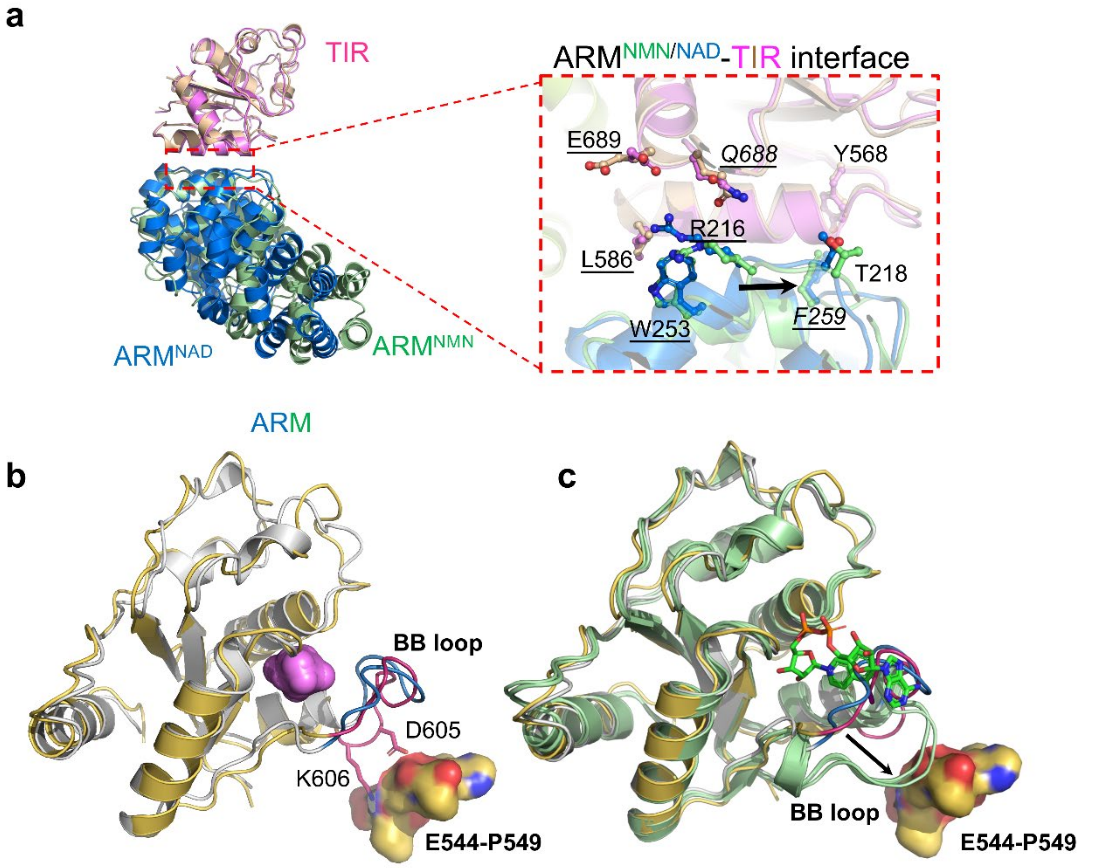

Supplementary Figure 10. The TIR:ARM interface and TIR conformation are restored in cryo-condition at presence of NAD or NMN. a Superposition of NMN-bound and NAD-bound SARM1 (PDB 7CM6) via the TIR domains. The TIR domains and ARM domains in both structures are shown as cartoon and the TIR-ARM interface is boxed and zoomed for detailed view. Some of the residues involved in TIR-ARM interactions are showed in ball-and-stick models. Compared to ARM^{NAD}, the helix where R216 and T218 locate moves rightward in ARM^{NMN}. **b** The superposition of the TIR domains in NMN- (yellow) and NAD-bound (grey, PDB 7ANW) SARM1. The BB loops in NMN-bound and NAD-bound SARM-1 are colored in magenta and blue, respectively. The active site of TIR domain is shown as a magenta ball. Two residues D605 and K606 in the BB loop are shown as ball-and-stick model, which are very close the C-terminus of the SAM domain (E544-P549, shown as surface model) and restrict the opening of the BB loop. **c** The superposition of TIR domains bound with NMN-bound (yellow), NAD (grey) and substrate mimetics (1AD, 2AD, 3AD, ara-F-ADPR, PDB 7NAG, 7NAH, 7NAI, 7NAJ and 7NAK, all in green). A black arrow indicates the opening of the BB loop upon substrate binding, which would clash with the SAM-TIR linker if TIR domain does not leave ARM domain.

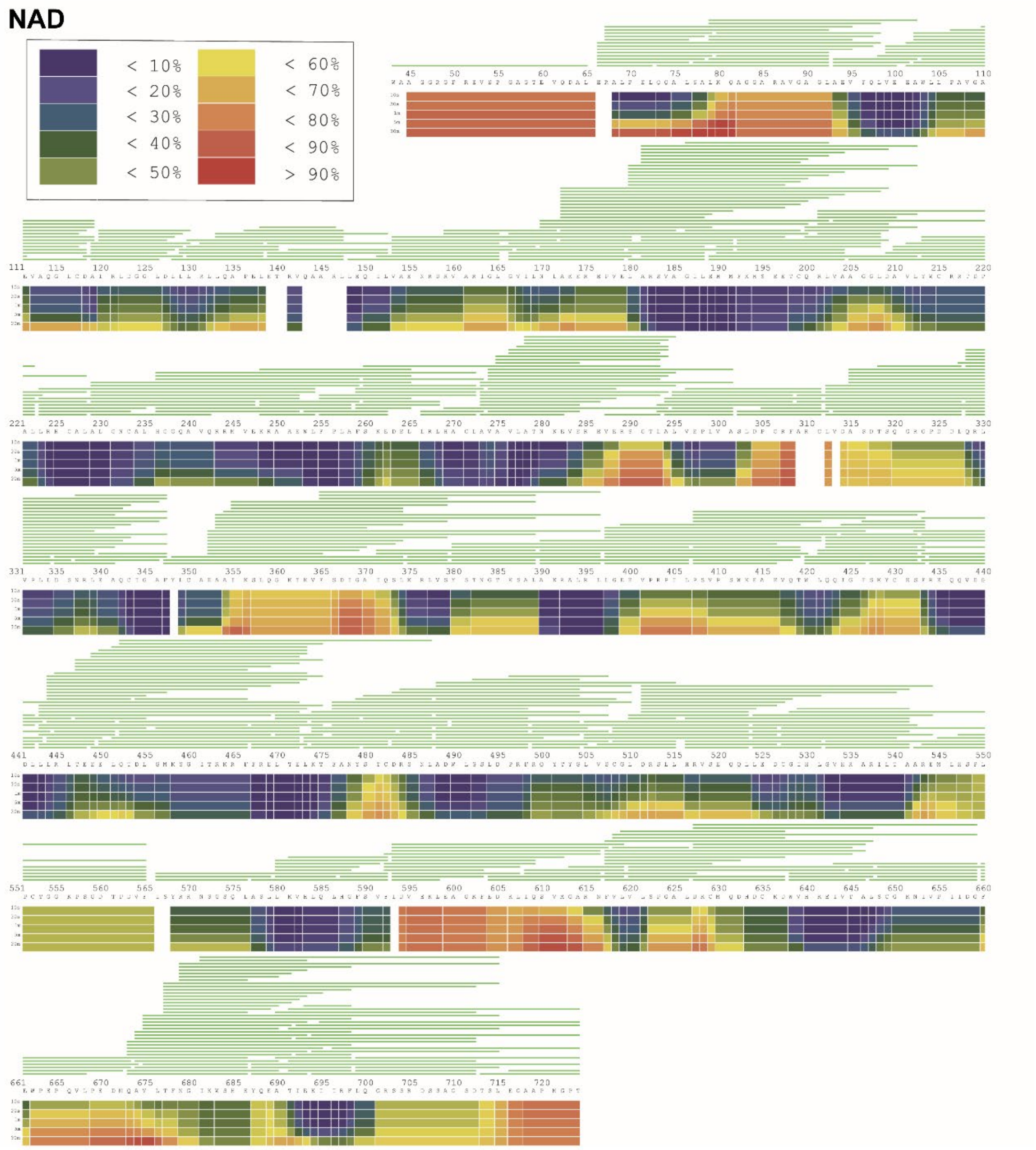
NAD
< 10%
< 20%
< 30%
< 40%
< 50%
< 60%
< 70%
< 80%
< 90%
> 90%

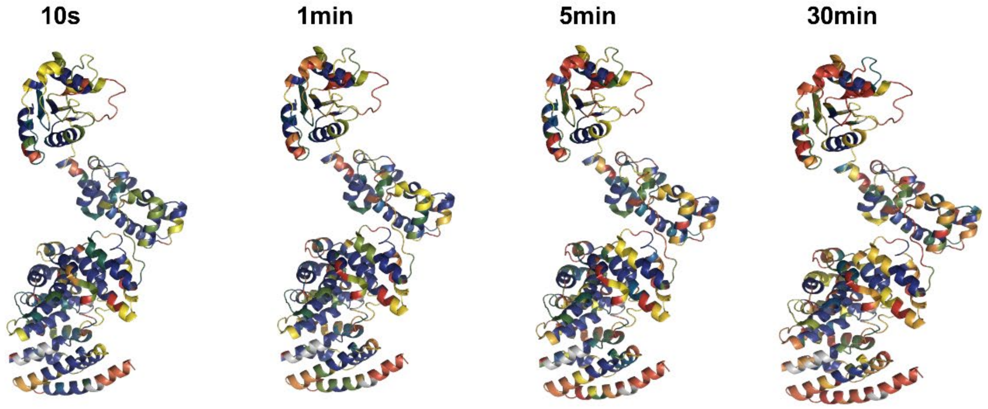
10s
1min
5min
30min

NMN

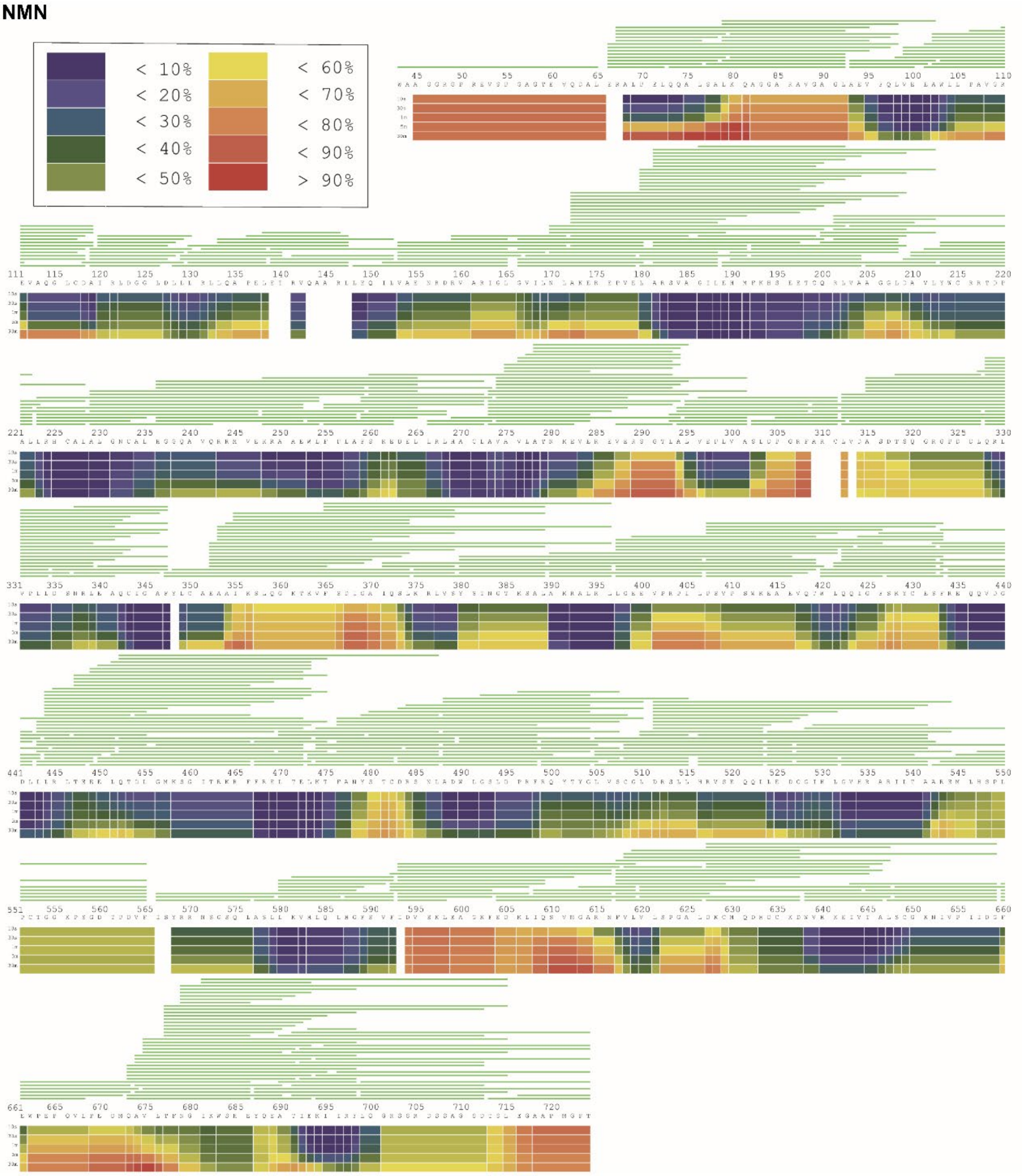

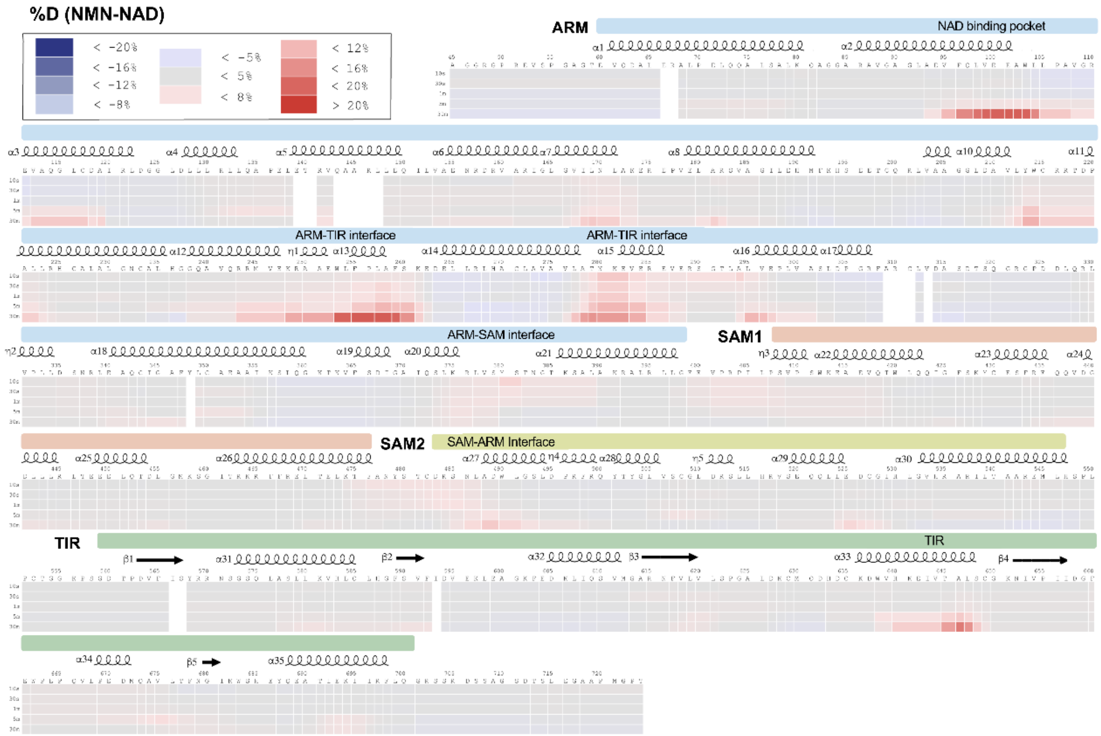

Supplementary Figure 11. Deuterium uptake data for full-length SARM1 in presence of NAD and NMN. HDX data for SARM1^{NAD} (top panel) and SARM1^{NMN} (middle panel) are shown in heatmap format. Absolute deuterium uptake after 10 s, 30 s, 1 min, 5 min and 30 min is indicated by a color gradient below the protein sequence. Peptide coverage is shown as green bars above the deuteration map. Cryo-EM of SARM1 (PDB 7CM6) with regions colored according to the same color code as in the heat map. Bottom panel is showing differences in deuterium uptake between NMN- and NAD-bound SARM1 (%D$^{(NMN)}$-%D$^{(NAD)}$). Protein secondary structure elements and domains are indicated above the sequence.

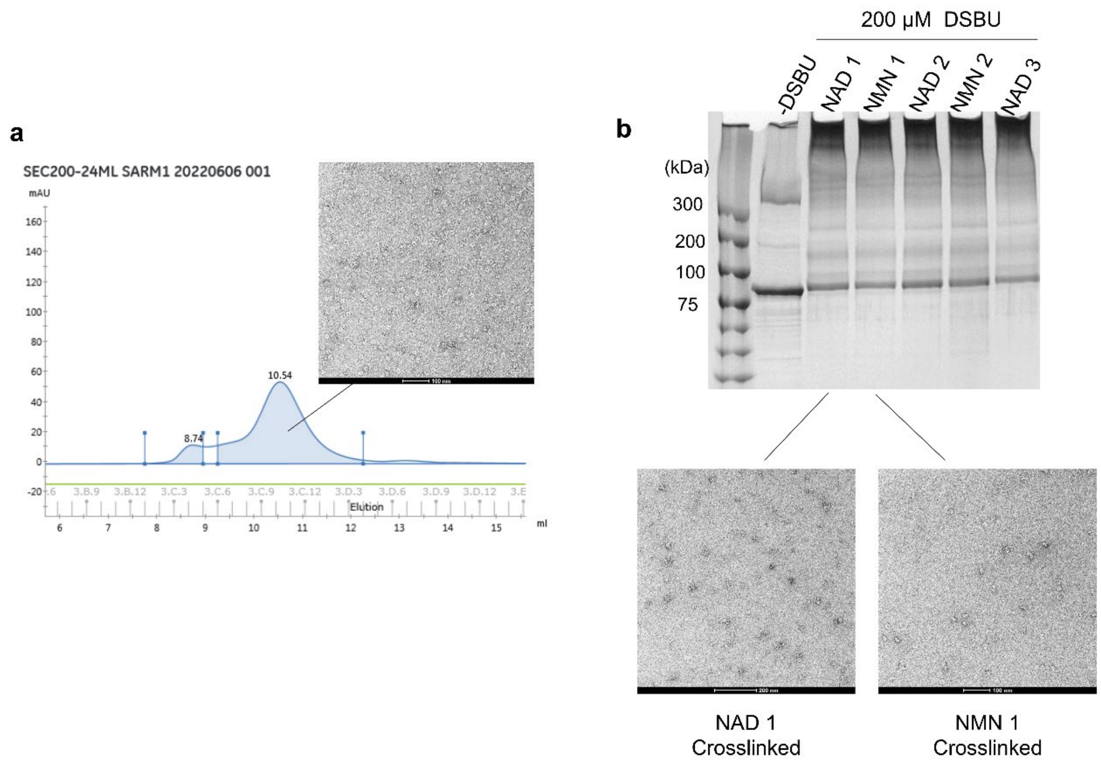

Supplementary Figure 12. SARM1 sample quality control and chemical crosslinking. Elution profile after size exclusion chromatography through Superdex 200 increase column, and negative staining EM analysis of purified SARM1 (**a**). SDS-PAGE and negative staining EM analysis of cross-linked SARM1 (**b**). Respective concentrations of SARM1 were 5 μM (NAD 1; NMN 1), 2.5 μM (NAD 2; NMN 2) and 1.25 μM (NAD 3) in both NAD and NMN condition. The data shown is the representative of 3 independent experiments.

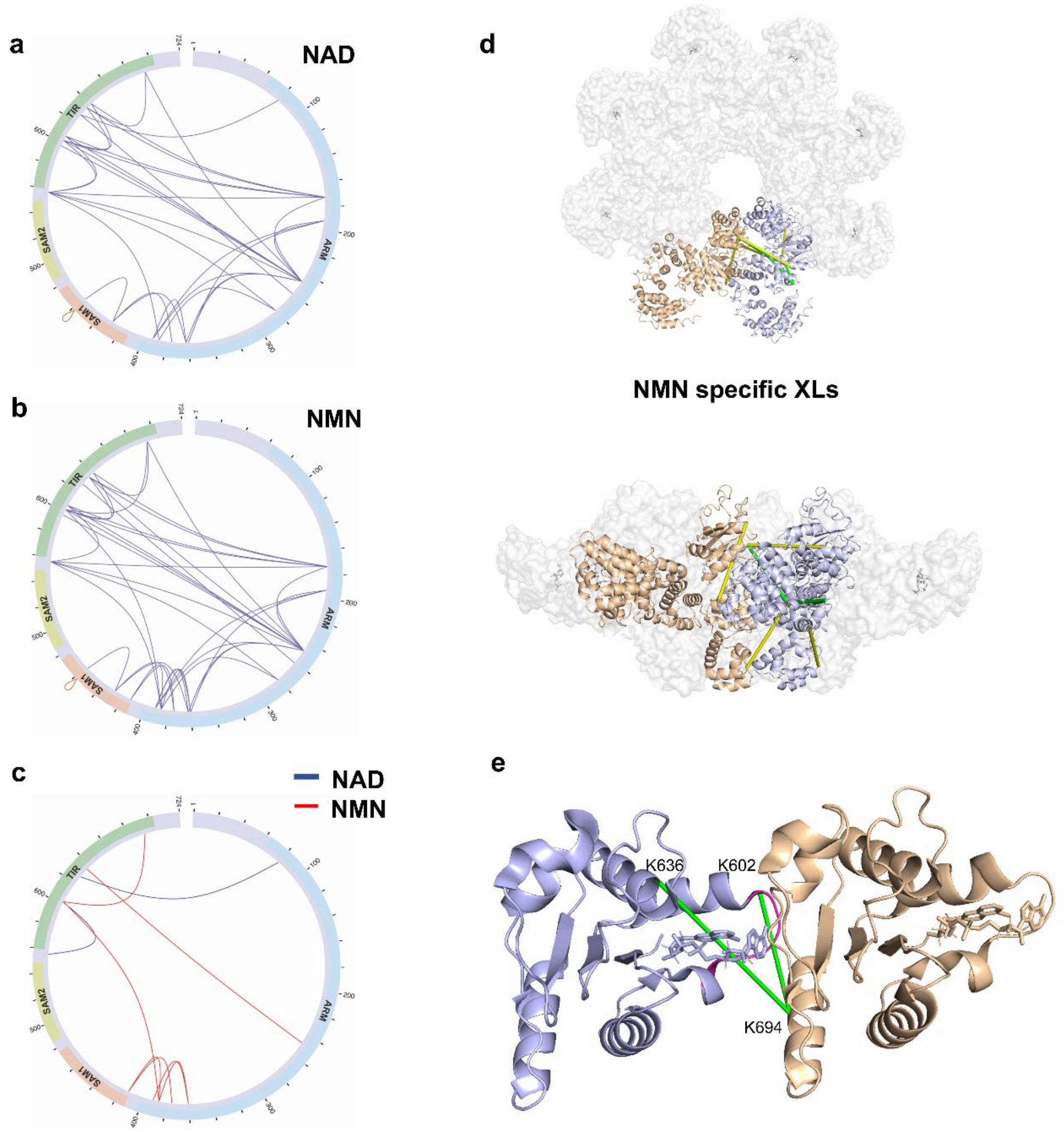

Supplementary Figure 13. XL-MS analysis of the SARM1 complex in presence of NAD and NMN. Circular plots displaying all the identified XLs for $SARM1^{NAD}$ (**a**), $SARM1^{NMN}$ (**b**) and NMN- or NAD- specific XLs (**c**). NMN-specific XLs mapped onto the structure of Nb-C6 complex. Satisfactory and unsatisfactory XLs are shown as green and yellow lines, respectively. **d** K694-K602 and K694-K636 XLs mapped onto the TIR domain homo-multimer (extracted from PDB 7NAK), with a BB loop (colored in pink) as the interface **(e)**.

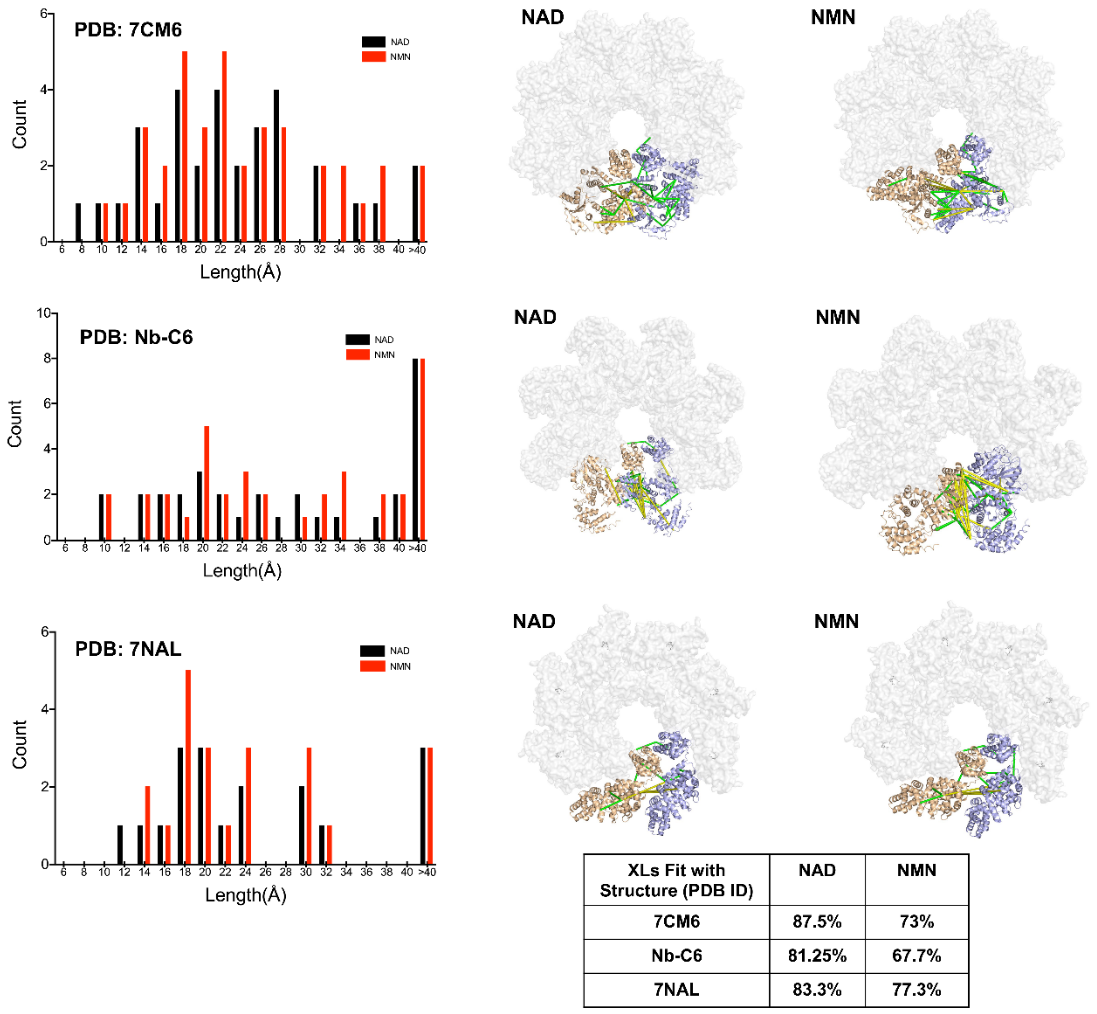

XLs Fit with Structure (PDB ID)	NAD	NMN
7CM6	87.5%	73%
Nb-C6	81.25%	67.7%
7NAL	83.3%	77.3%

Supplementary Figure 14. XL-MS analysis of the SARM1 complex in presence of NAD and NMN.

Distance analysis of all the identified XLs for SARM1^{NAD} and SARM1^{NMN} when mapped onto the cryo-EM structures of: Nb-C6, SARM1^{NAD} (PDB 7CM6), and ARM-SAM^{NMN} (PDB 7NAL). Satisfactory and unsatisfactory XLs are shown as green and yellow lines, respectively. The table is showing percentage of satisfied XLs of residues with distances <30 Å and without steric clashes, after mapping onto the protein structures.

Supplementary Table 1. HDX data statistics

Data Set	NAD (control)	NMN
HDX reaction details	Final D_2O concentration during labeling reaction 90% ,<br>25 mM HEPES, 150 mM NaCl, pHread 8.0, 28°C	Final D_2O concentration during labeling reaction 90% , 25 mM HEPES, 150 mM NaCl, pHread 8.0, 28°C
HDX time course (sec)	10, 30, 60, 300, 1800	10, 30, 60, 300, 1800
HDX control samples	none	none
Back-exchange (mean / IQR over entire project)	unknown	
# of peptides	557	557
Sequence coverage	0.942	0.942
Average peptide length / Redundancy	18.39 / 14.15	18.39 / 14.15
Replicates	3	3
Repeatability (avg. stddev of #D)	0.0642	0.0609
Significant differences in HDX (delta HDX > X D) (99% CI)	0.4343 D	
Data processing software	HDExaminer 3.3 (Sierra Analytics)	

Supplementary Table 2. Cryo-EM data collection, processing and modeling

**Data Collection**			
Microscope	FEI TITAN KROIS		
Imaging Mode	Microprobe (EF)		
Detector	Gatan K2 Summit		
Voltage (kV)	300		
Magnification	130,000 x		
Pixel Size (Å /pixel)	1.076		
Total electron dose (e-/Å2)	50		
Frame Rate (frames/second)	6.3		
Defocus range (μm)	-1.0 ~ -2.5		
Micrographs collected	4,605		
**Processing**			
Map Description	Overall SARM1/NMN/Nb-C6 Structure	Local-refined TIR-ARM monomer, conformation 1	Local-refined TIR-ARM monomer, conformation 2
EMDB Reference Number	EMD-34198	EMD-34165	EMD-34166
Softwares	cisTEM-1.0.0,	cisTEM-1.0.0,	cisTEM-1.0.0,
Total Extracted Particles	Relion-3.1.2	Relion-3.1.2	Relion-3.1.2
Particles for Symmetry Expansion		3,089,111	3,089,111
Particles for Multibody Refinement		298,862	133,097
Particles for Final Reconstruction	208,299	298,862	133,097
Refinement Approach	3D Refinement & Reconstruction	Multi-body Refinement	Multi-body Refinement
Map Symmetry	C8	C1	C1
Map Sharpening B Factors	90.00	134.313	112.232
Map Resolution	2.76	3.74	3.78
FSC Threshold	0.143	0.143	0.143
**Modeling**			
PDB Reference Number	PDB-8GQ5	PDB-8GNI	PDB-8GNJ
Softwares	Coot-0.9, Phenix-1.16	Coot-0.9, Phenix-1.16	Coot-0.9, Phenix-1.16
Initial Models	PDB-7DJT, PDB-5F1K	PDB-7DJT, PDB-5F1K	PDB-7DJT, PDB-5F1K
Model Resolution	3.0	3.5	3.5
Atoms	42592	5832	5832
Ligands	0	1 (NMN)	1 (NMN)
**Validation**			
MolProbity score	1.75	1.91	1.91
All-atom clashscore	6.28	10.15	11.77
Rotamers outliers (%)	0.00	0.00	0.00
CBeta Outliers(%)	0.00	0.00	0.00
CC(Mask)	0.86	0.75	0.81
Mean CC for Ligands		0.71	0.85
R.M.S deviations			
Bonds lengths (Å)	0.010	0.005	0.011
Bonds angles (°)	1.094	1.022	1.325
Ramachandran plot statistics			
Preferred (%)	94.03	94.12	95.41
Allowed (%)	5.97	5.68	4.59
Outlier (%)	0.00	0.00	0.00
