# Supplementary material for: A conformation-specific nanobody targeting the nicotinamide mononucleotide-activated state of SARM1
Source: Nat Commun. 2022 Dec 22;13:7898. doi: 10.1038/s41467-022-35581-y (PMC9780360; doi:10.1038/s41467-022-35581-y)
Supplement: Supplementary file 2 — Description of Additional Supplementary Files [file 41467_2022_35581_MOESM2_ESM.docx]

File Name: Supplementary Data 1

Description: SARM1 HDX-MS peptides with %D values

File Name: Supplementary Data 2

Description: SARM1 XL-MS peptides

File Name: Supplementary Data3

Description: D317R/S319Y/Q320Y modelling based on 7ANW

File Name: Supplementary Data4

Description: R216Q/L257C modelling based on 7ANW
