# Supplementary material for: A conformation-specific nanobody targeting the nicotinamide mononucleotide-activated state of SARM1
Source: Nat Commun. 2022 Dec 22;13:7898. doi: 10.1038/s41467-022-35581-y (PMC9780360; doi:10.1038/s41467-022-35581-y)
Supplement: Supplementary file 7 — Reporting Summary [file 41467_2022_35581_MOESM7_ESM.pdf]

nature portfolio

Corresponding author(s): Professor Yongjuan Zhao (zhaoyongjuan@cuhk.edu.cn) (The Chinese University of Hongkong, Shenzhen)

Last updated by author(s): 2022/11/28

Reporting Summary

Nature Portfolio wishes to improve the reproducibility of the work that we publish. This form provides structure for consistency and transparency in reporting. For further information on Nature Portfolio policies, see our Editorial Policies and the Editorial Policy Checklist.

Statistics

For all statistical analyses, confirm that the following items are present in the figure legend, table legend, main text, or Methods section.

n/a	Confirmed	
☐	☒	The exact sample size (n) for each experimental group/condition, given as a discrete number and unit of measurement
☐	☒	A statement on whether measurements were taken from distinct samples or whether the same sample was measured repeatedly
☐	☒	The statistical test(s) used AND whether they are one- or two-sided<br>*Only common tests should be described solely by name; describe more complex techniques in the Methods section.*
☒	☐	A description of all covariates tested
☒	☐	A description of any assumptions or corrections, such as tests of normality and adjustment for multiple comparisons
☐	☒	A full description of the statistical parameters including central tendency (e.g. means) or other basic estimates (e.g. regression coefficient) AND variation (e.g. standard deviation) or associated estimates of uncertainty (e.g. confidence intervals)
☐	☒	For null hypothesis testing, the test statistic (e.g. F, t, r) with confidence intervals, effect sizes, degrees of freedom and P value noted<br>*Give P values as exact values whenever suitable.*
☒	☐	For Bayesian analysis, information on the choice of priors and Markov chain Monte Carlo settings
☒	☐	For hierarchical and complex designs, identification of the appropriate level for tests and full reporting of outcomes
☐	☒	Estimates of effect sizes (e.g. Cohen's d, Pearson's r), indicating how they were calculated

Our web collection on statistics for biologists contains articles on many of the points above.

Software and code

Policy information about availability of computer code

Data collection	Cryo-EM data was collected using Leginon Version 3,Serial EM 3.6.21.<br>Fluorescent image was captured by NIS-Elements AR analysis 5.01.<br>SPR data was collected using BIAcore Insight 3.0.12<br>Confocal image was acuquired by NIS-Elements 5.6.0
Data analysis	Cryo-EM data was processed using Relion 3.1, CTFFIND4 v4.1.2, Coot 0.9, Pymol 2.3.4, Chimera 1.12,Phenix 1.16..<br>Fluorescent image was analysis by NIS-Elements AR analysis 5.01<br>Crosslinking experiments were analysis by Proteome Discoverer 2.5, Xlinf software 2.5.<br>HDX-MS experiments was analysis by HDExaminer v3.3.0.<br>SPR data was processed by BIAcore Insight evaluation software 3.0.12.<br>Statistical analysis was done by GraphPad Prism 8.0.1.<br>Co-localization analysis was processed by ImageJ 1.53 and Imaris Bitplane 7.4.2

For manuscripts utilizing custom algorithms or software that are central to the research but not yet described in published literature, software must be made available to editors and reviewers. We strongly encourage code deposition in a community repository (e.g. GitHub). See the Nature Portfolio guidelines for submitting code & software for further information.

Data

Policy information about availability of data

All manuscripts must include a data availability statement. This statement should provide the following information, where applicable:

- Accession codes, unique identifiers, or web links for publicly available datasets
- A description of any restrictions on data availability
- For clinical datasets or third party data, please ensure that the statement adheres to our policy

Atomic coordinates for SARM1/NMN/Nanobody-C6 have been deposited in the wwPDB under accession codes 8GQ5 [https://doi.org/10.2210/pdb8GQ5/pdb] (Overall Structure), 8GNI [https://doi.org/10.2210/pdb8GNI/pdb] (TIR-ARM Conformation 1), and 8GNJ [https://doi.org/10.2210/pdb8GNJ/pdb] (TIR-ARM Conformation 2), respectively. Cryo-EM densities have been deposited in the Electron Microscopy Data Bank (EMDB) under accession codes EMD-34198 [https://www.ebi.ac.uk/pdbe/entry/emdb/EMD-34198] (Overall Structure), EMD-34165 [https://www.ebi.ac.uk/pdbe/entry/emdb/EMD-34165] (TIR-ARM Conformation 1), and EMD-34166 [https://www.ebi.ac.uk/pdbe/entry/emdb/EMD-34166] (TIR-ARM Conformation 2), respectively. The mass spectrometry proteomics data have been deposited to the ProteomeXchange Consortium via the PRIDE46 partner repository with the dataset identifier PXD033528 [https://www.ebi.ac.uk/pride/archive/projects/PXD033528]. Source data are provided with this paper.

Field-specific reporting

Please select the one below that is the best fit for your research. If you are not sure, read the appropriate sections before making your selection.

[x] Life sciences   [ ] Behavioural & social sciences   [ ] Ecological, evolutionary & environmental sciences

For a reference copy of the document with all sections, see nature.com/documents/nr-reporting-summary-flat.pdf

Life sciences study design

All studies must disclose on these points even when the disclosure is negative.

Sample size	There was no statistical method to predetermine sample size.<br>The amount of protein samples involving the biochemical experiments was based on our previous research or commonly used sample size in the field of study.<br>For cryo-EM, 4,605 movies were collected, and 3,089,111 particles were used for the final refinement. The sample size was determined by available microscope time and movies were sufficient for atomic model building and structure refinement.<br>For HDX-MS,557 peptides common to both NAD- and NMN-liganded SARM1 were chosen for data analysis. The sample size was determined by the resolution of the MS, and the peptide generated covered more than 95% of the proteins which is sufficient for analysis.
Data exclusions	Cryo-EM micrographs were inspected manually and the ones with bad ice or large drift were discarded. Also, some particles which were false-picked, broken, damaged were sorted out in the procedure.<br>In HDX-MS, the low quality peptides or spectra wrongly identified were removed from the analysis. Because low intensity peptide signal was messed with background noise, causing false %D rate.
Replication	The number of the replications is stated in the Figures Legends, and all experiments were replicated in at least 3 independent experiments.<br>All our attempts at replication were successful.
Randomization	For the quantification of cell fluorescence, we randomly selected the areas and the cells exposed to the 405nm laser light.<br>For other experiments in this study, no randomization was applied due to the nature of the experiments.
Blinding	Blinding was not relevant to this study, because no human subjects or animals were involved or subjective allocation was involved.

Reporting for specific materials, systems and methods

We require information from authors about some types of materials, experimental systems and methods used in many studies. Here, indicate whether each material, system or method listed is relevant to your study. If you are not sure if a list item applies to your research, read the appropriate section before selecting a response.

Materials & experimental systems

n/a	Involved in the study
☐	☒ Antibodies
☐	☒ Eukaryotic cell lines
☒	☐ Palaeontology and archaeology
☐	☒ Animals and other organisms
☒	☐ Human research participants
☒	☐ Clinical data
☒	☐ Dual use research of concern

Methods

n/a	Involved in the study
☒	☐ ChIP-seq
☒	☐ Flow cytometry
☒	☐ MRI-based neuroimaging

Antibodies

Antibodies used

Mouse anti-His6 (TransGen Biotech HT501-01,1:1000)
Mouse anti-Tom20(Santa Cruz sc-17764,1:1000),
Mouse anti-GFP (TransGen Biotech HT801-01,1:1000)
Mouse anti-HSP90 (abcam ab59459, 1:2000)
Rabbit anti-tubulin (abcam ab184966, 1:2000)
HRP/Anti-M13 Monoclonal (GE lifesciences GE27-9421-01, 1:1000)
Anti-mouse IgG, HRP-linked Antibody(CST #7076, 1:2000)
Anti-rabbit IgG, HRP-linked Antibody (CST #7074, 1:2000)
Alexa Fluor 568-conjugated donkey anti-rabbit IgG (Thermo Fisher A10042, 1:1000)
Alexa Fluor 647-conjugated donkey anti-mouse IgG (Thermo Fisher A31571, 1:1000)

Rabbit anti-SARM1 (1:1000)and CD38 nanobody Nb-1053 (concentration in Figure Legend)was purified by our lab.

Validation

All antibodies(expect anti-SARM1) used in this study are commercially available and have been widely used in publications . They have been validated for the western blot or studies and immunofluorescence specificity for cells. The complete information and validation details are provided in the respective manufacturer's website data-sheets.

Mouse anti-His6 (https://www.transgenbiotech.com/tag_antibody/proteinfind_anti_his_mouse_monoclonal_antibody.html)
Mouse anti-Tom20(https://www.scbt.com/p/tom20-antibody-f-10),
Mouse anti-GFP (https://www.transgenbiotech.com/tag_antibody/proteinfind_anti_gfp_mouse_monoclonal_antibody.html)
Mouse anti-HSP90 (https://www.abcam.cn/hsp90-antibody-d7a-ab59459.html)
Rabbit anti-tubulin (https://www.abcam.cn/tubulin-antibody-epr13798-loading-control-ab184966.html)
HRP/Anti-M13 Monoclonal (https://www.sigmaaldrich.com/US/en/product/sigma/ge27942101?gclid=Cj0KCQiA1ZGcBhCoARIsAGQ0kkpZ0yUQQCR5O3sZM_vgfyhK0kxB1m7q1DY5GQggE9jmIXgKF30lfqkaAofhEALw_wcB&gclsrc=aw.ds)
Anti-mouse IgG, HRP-linked Antibody(https://en.cellsignal.jp/products/secondary-antibodies/anti-mouse-igg-hrp-linked-antibody/7076)
Anti-rabbit IgG, HRP-linked Antibody (https://en.cellsignal.jp/products/secondary-antibodies/anti-rabbit-igg-hrp-linked-antibody/7074)
Alexa Fluor 568-conjugated donkey anti-rabbit IgG (https://www.thermofisher.cn/cn/zh/antibody/product/Donkey-anti-Rabbit-IgG-H-L-Highly-Cross-Adsorbed-Secondary-Antibody-Polyclonal/A10042)
Alexa Fluor 647-conjugated donkey anti-mouse IgG (https://www.thermofisher.cn/cn/zh/antibody/product/Donkey-anti-Mouse-IgG-H-L-Highly-Cross-Adsorbed-Secondary-Antibody-Polyclonal/A-31571)

The anti-SARM1 was validated and optimized for western bolting by our lab, and have published before.
https://doi.org/10.7554/eLife.67381

Eukaryotic cell lines

Policy information about cell lines

Cell line source(s)	Expi293F cells(Sino Biological Inc.) HEK293(ATCC #CRL-1573), HEK293T(ATCC #CRL-11268)
Authentication	None of the cell lines used were authenticated.
Mycoplasma contamination	The cell lines were not tested for mycoplasma contamination.
Commonly misidentified lines (See ICLAC register)	No commonly misidentified cell lines were used.

Animals and other organisms

Policy information about studies involving animals; ARRIVE guidelines recommended for reporting animal research

Laboratory animals	A healthy, adult, male Alpaca were raised and maintained by AlpaLife Inc.
Wild animals	The study did not involve wild animals.
Field-collected samples	The study did not involve samples collected from the field.
Ethics oversight	All study involving alpaca raising or immunizations at Shenzhen KangTi Life Technology Co., Ltd (AlpaLife) complies with the relevant ethical regulations for animal testing and research.

Note that full information on the approval of the study protocol must also be provided in the manuscript.
