# Supplementary material for: A conformation-specific nanobody targeting the nicotinamide mononucleotide-activated state of SARM1
Source: Nat Commun. 2022 Dec 22;13:7898. doi: 10.1038/s41467-022-35581-y (PMC9780360; doi:10.1038/s41467-022-35581-y)
Supplement: Supplementary file 8 — Source Data [file 41467_2022_35581_MOESM8_ESM.zip › NCOMMS-22-14729C_sd2/Source Data/Fig 1C.pdf]

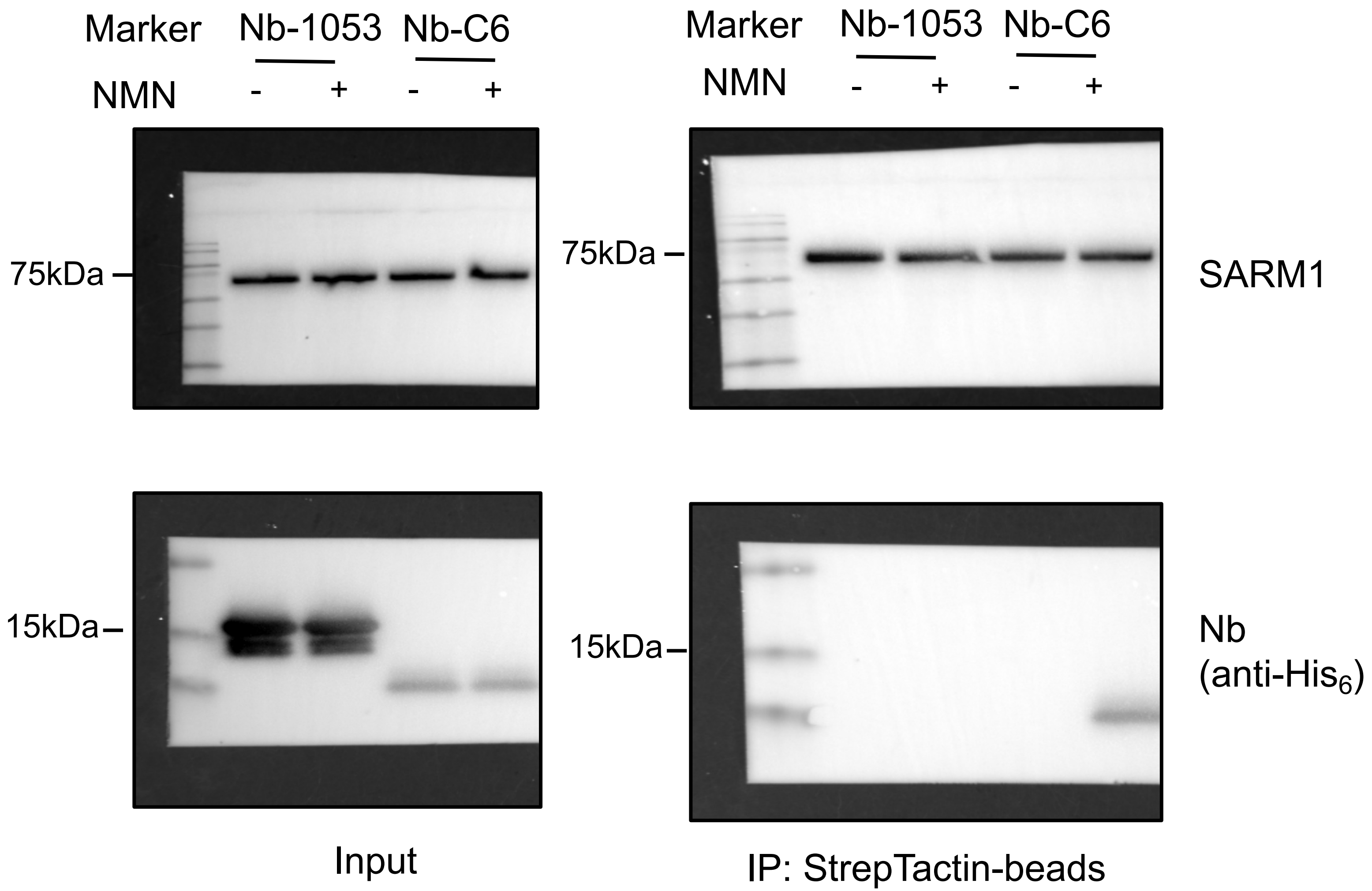

Nb-C6 binds to the NMN-activated SARM1 determined by the pulldown assay. The cell lysates containing the recombinant SARM1 were incubated with the StrepTactinTM beads, together with 200 ng/mL Nb-C6, or a CD38 nanobody Nb-105326 as a control,in presence or absence of 100 µM NMN. The protein complex was eluted by 2 mM biotin and analyzed by western blots.
