# Supplementary figures and images for: A conformation-specific nanobody targeting the nicotinamide mononucleotide-activated state of SARM1
Source: Nat Commun. 2022 Dec 22;13:7898. doi: 10.1038/s41467-022-35581-y (PMC9780360; doi:10.1038/s41467-022-35581-y)

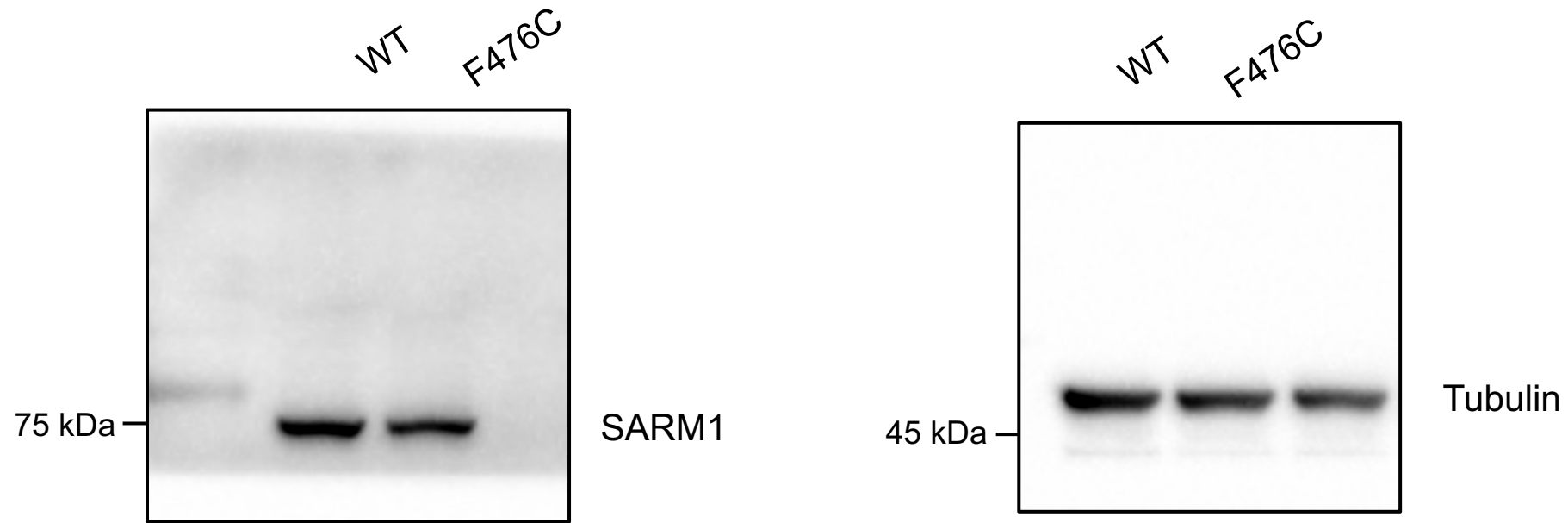

The activity and NMN-responsiveness of the SARM1 mutants measured

Supplement: Supplementary file 8 — Source Data [file 41467_2022_35581_MOESM8_ESM.zip › NCOMMS-22-14729C_sd2/Source Data/Fig 5b Western.pdf]

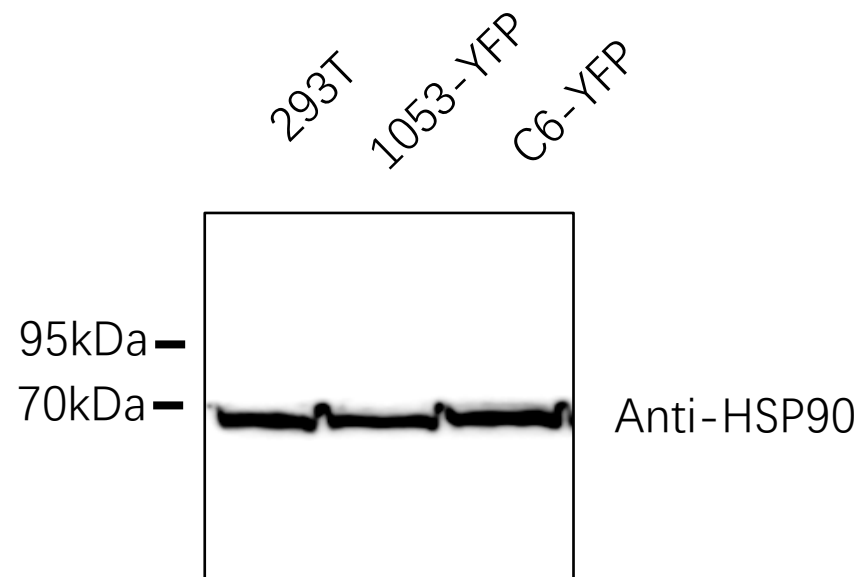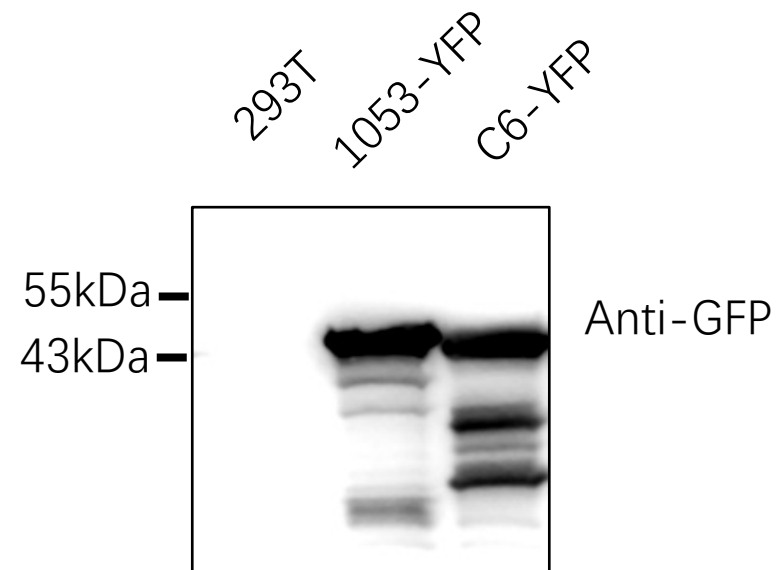

Supplement: Supplementary file 8 — Source Data [file 41467_2022_35581_MOESM8_ESM.zip › NCOMMS-22-14729C_sd2/Source Data/Fig S2f.pdf]

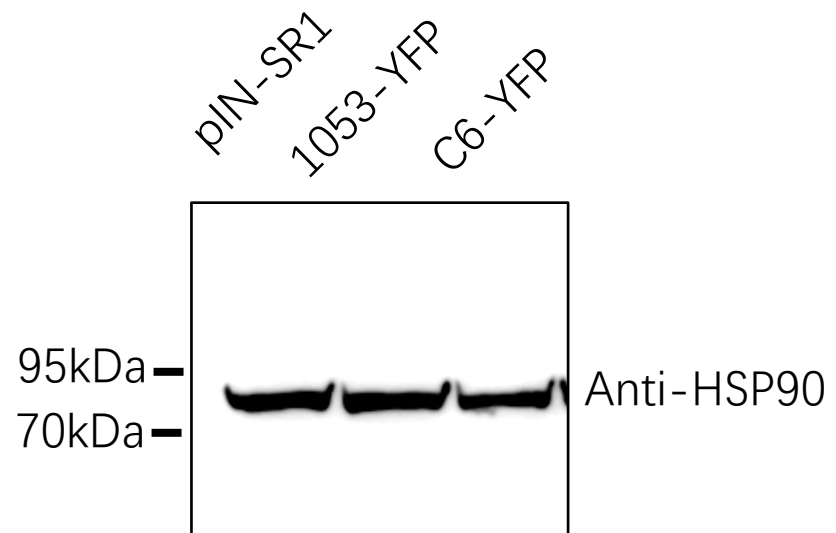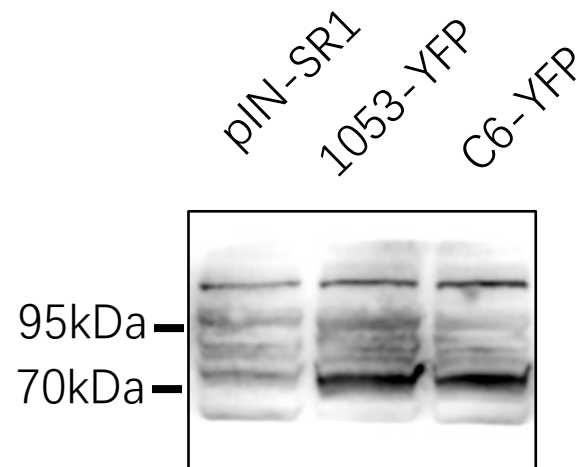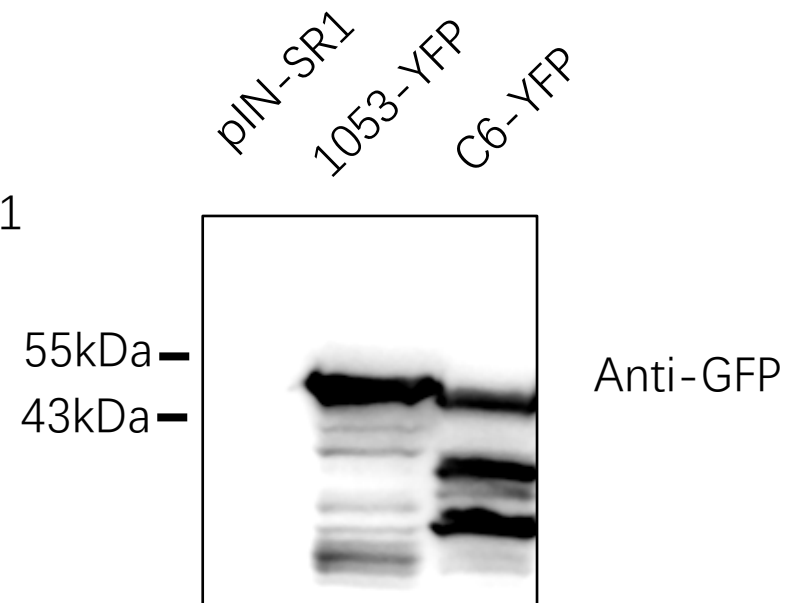

Supplement: Supplementary file 8 — Source Data [file 41467_2022_35581_MOESM8_ESM.zip › NCOMMS-22-14729C_sd2/Source Data/Fig S2g.pdf]

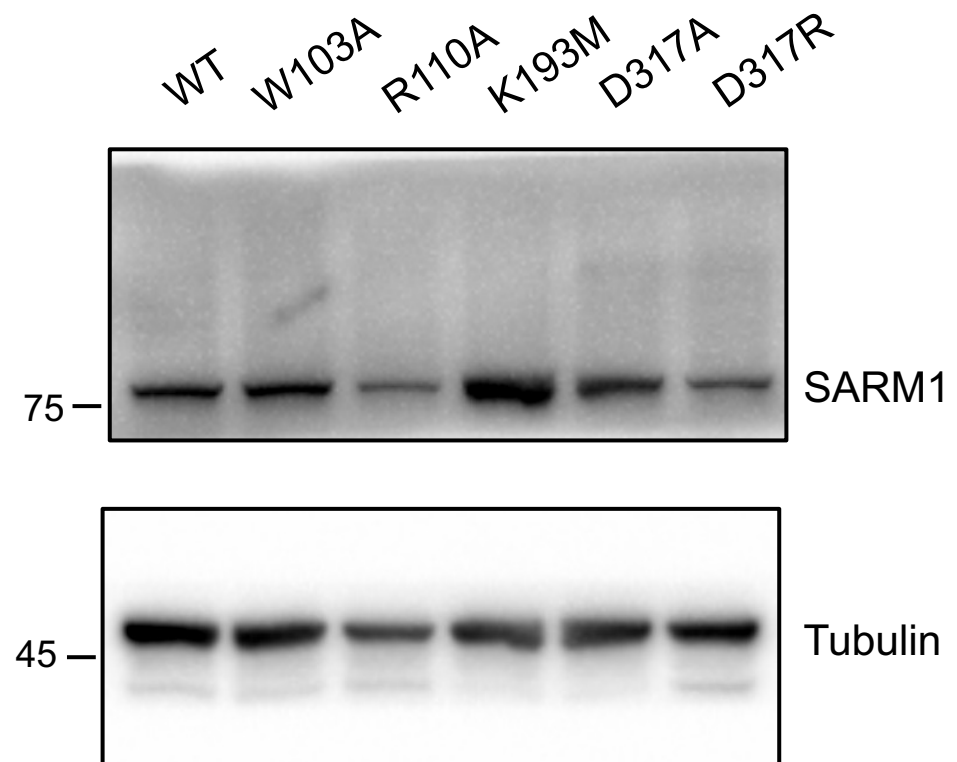

Supplement: Supplementary file 8 — Source Data [file 41467_2022_35581_MOESM8_ESM.zip › NCOMMS-22-14729C_sd2/Source Data/Fig S8a.pdf]

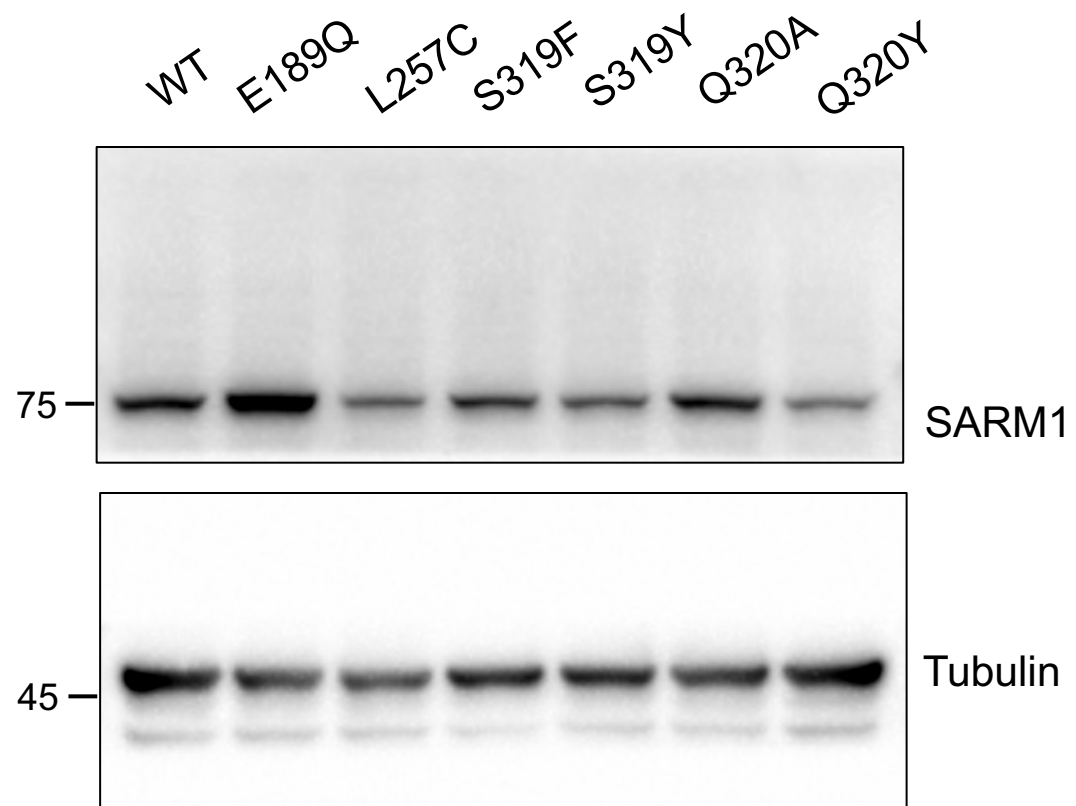

Supplement: Supplementary file 8 — Source Data [file 41467_2022_35581_MOESM8_ESM.zip › NCOMMS-22-14729C_sd2/Source Data/Fig S8c.pdf]
